# Genetic variation of dynamic fiber elongation and developmental quantitative trait locus mapping of fiber length in upland cotton (*Gossypium hirsutum* L.)

**DOI:** 10.1186/s12864-018-5309-2

**Published:** 2018-12-06

**Authors:** Jianjiang Ma, Yanhui Geng, Wenfeng Pei, Man Wu, Xingli Li, Guoyuan Liu, Dan Li, Qifeng Ma, XinShan Zang, Shuxun Yu, Jinfa Zhang, Jiwen Yu

**Affiliations:** 1 College of Agronomy, Northwest A&F University, Yangling, 712100 Shanxi China; 2 State Key Laboratory of Cotton Biology, Cotton Institute of the Chinese Academy of Agricultural Sciences, Key Laboratory of Cotton Genetic Improvement, Ministry of Agriculture, Anyang, 455000 Henan China; 3 Department of Plant and Environmental Sciences, New Mexico State University, Las Cruces, 880033 USA

**Keywords:** *Gossypium hirsutum*, GWAS, Dynamic fiber length, Transcriptome

## Abstract

**Background:**

In upland cotton (*Gossypium hirsutum* L.), genotypes with the same mature fiber length (FL) might possess different genes and exhibit differential expression of genes related to fiber elongation at different fiber developmental stages. However, there is a lack of information on the genetic variation influencing fiber length and its quantitative trait loci (QTLs) during the fiber elongation stage. In this study, a subset of upland cotton accessions was selected based on a previous GWAS conducted in China and grown in multiple environments to determine the dynamic fiber length at 10, 15, 20, and 25 days post-anthesis (DPA) and maturity. The germplasm lines were genotyped with the Cotton 63 K Illumina single-nucleotide polymorphism (SNP) array for GWAS.

**Results:**

A total of 25, 38, 57, 89 and 88 SNPs showed significant correlations with fiber length at 10, 15, 20 and 25 DPA and maturity, respectively. In addition, 60 more promising SNPs were detected in at least two tests and two FL developmental time points, and 20 SNPs were located within the confidence intervals of QTLs identified in previous studies. The fastest fiber-length growth rates were obtained at 10 to 15 DPA in 69 upland cotton lines and at 15 to 20 DPA in 14 upland cotton accessions, and 10 SNPs showed significant correlations with the fiber-length growth rate. A combined transcriptome and qRT-PCR analysis revealed that two genes (D10G1008 and D13G2037) showed differential expression between two long-fiber genotypes and two short-fiber genotypes.

**Conclusions:**

This study provides important new insights into the genetic basis of the time-dependent fiber-length trait and reveals candidate SNPs and genes for improving fiber length in upland cotton.

**Electronic supplementary material:**

The online version of this article (10.1186/s12864-018-5309-2) contains supplementary material, which is available to authorized users.

## Background

Cotton is among the most economically important crops worldwide and provides large amounts of natural fiber to the textile industry. There are four cultivated cotton species: two diploid species (*Gossypium herbaceum* L. and *G. arboreum* L.) and two tetraploid species (*G. hirsutum* L. and *G. barbadense* L.) [1, 2]. Upland cotton (*G. hirsutum* L.) accounts for more than 95% of cotton production worldwide [3]. Another cultivated tetraploid species, referred to as Egyptian, Pima or Sea-Island cotton (*G. barbadense* L.), exhibits superior extra-long fibers [2]. Cotton fibers are the longest and fastest-growing cells in plants, and each cotton fiber consists of a single cell generated from the epidermal layer of the ovule (seed). Cotton fiber development mainly comprises four distinct, but overlapping stages: initiation, fast elongation, secondary wall thickening and maturity [4, 5]. The elongation of fibers occurs after initiation and lasts until 25–30 days post anthesis (DPA), which determines the ultimate fiber length [6–8]. It is known that quantitative traits such FL are affected by genotype (G), environment (E) and genotype by environment interactions (G × E). For example, significant E and G × E were reported for FL by Huang et al. who tested 503 upland cotton accessions in eight environments [9] and by Sun et al. who tested 719 upland cotton accessions in eight environments [10]. However, numerous genes are found preferentially and differentially expressed during fiber development [11–13]. Therefore, genotypes with the same final fiber length might have different genes and gene expression profiles during fiber elongation. The pyramiding of these different fiber genes from the genotypes with the same fiber length might further enhance fiber length in new breeding lines. However, the genetic basis of dynamic fiber elongation in cotton is currently unknown.

FL is among the most important fiber-quality traits, and the selection of long-fiber cultivars is therefore important for the textile industry. Mature FL is a complex trait controlled by a multitude of quantitative trait loci (QTLs) [14–16]. Previous studies have dissected the genetic architecture of FL through traditional QTL linkage mapping using bi-parental populations, and approximately 120 QTLs for FL traits have been identified [16–20]. However, most of the QTLs obtained from interspecific populations are not directly applicable to upland cotton improvement because they are localized in very large genetic regions, and most are not stable across different populations; moreover, the molecular mechanisms underlying the QTLs are unclear. In contrast to QTL linkage mapping, genome-wide association studies (GWAS) based on linkage disequilibrium (LD) can effectively associate genotypes with phenotypes in a natural population and can simultaneously detect many natural allelic variations. With the development of technologies for sequencing cotton genomes, GWAS has been successfully applied to the genetic dissection of fiber quality traits, including FL. Based on the resequencing of markers, seven and three FL QTLs were identified in 318 and 352 diverse *G. hirsutum* accessions, respectively [21, 22]. In addition, 503 and 719 diverse *G. hirsutum* accessions were individually genotyped using the Cotton 63 K Illumina single nucleotide polymorphism (SNP) array, resulting in the identification of 11 and 20 significant SNPs, respectively, associated with the FL trait [9, 10].

Because almost all of the FL QTLs identified to date in cotton are for mature fibers, it is currently unknown when and how these QTLs act during the fiber elongation stage. An analysis of phenotypic data at multiple developmental stages can aid the identification of stage-specific QTLs and consistent QTLs across stages for the trait of interest, such as FL. Furthermore, such an approach might uncover new QTLs that were not previously identified at the harvest stage. In fact, QTLs related to important agronomic traits in cotton, such as boll number, plant height and flowering timing, have been detected at different developmental time points [23, 24]. However, the phenotypic value of FL is normally measured at the maturity stage, which ignores the dynamic elongation process of FL with respect to gene-specific expression at different fiber developmental time points. For example, some genes related to fiber development, such as GhPK6, GhJAZ2 and GhCaM7-like, are selectively expressed during different fiber-growth periods [11–13]. Understanding the genetic mechanisms underlying FL at different developmental time points will aid the elucidation of the mechanism of fiber elongation. Currently, there is a lack of work in reporting QTLs for fiber length at different fiber developmental stages and growth rate in fiber length.

Previous GWAS reports used approximately 300–700 diverse upland cotton accessions from China [9, 10, 22, 25, 26]. Based on the mature FL data of the most of the upland cotton accessions from public databases and our lab, we selected 83 representative upland cotton lines with a wide range variation of mature FL from different sources or pedigrees. The accession number would allow sampling developing fibers in multiple timepoints during fiber development, because the task in harvesting ovules and collecting developing fibers is a time-consuming and laborious process. The accessions were grown at a single, representative cotton production location (Anyang, Henan province) of the Yellow River Valley for 3 years (2014, 2015 and 2016) to measure FL at five different fiber developmental time points (10, 15, 20, and 25 DPA and maturity). The genotypes of the accessions were determined using the Cotton 63 K Illumina SNP array [27]. The aim of this study was to detect dynamic QTLs for FL in upland cotton.

## Methods

### Plant materials

A total of 83 upland cotton lines were selected based on a previous GWAS involving approximately 300–700 diverse upland cotton accessions from China, and the seeds were obtained from the National Cotton Germplasm Collections of the Low-temperature Germplasm Gene Bank of the Institute of Cotton Research, Chinese Academy of Agricultural Sciences (CRI-CAAS) (Additional file 1: Table S1). Ten *G. barbadense* lines were used as an outgroup (Additional file 1: Table S2). All 93 cotton accessions were grown in Anyang (Henan province, 36.06°N, 114.49°E) in 2014, 2015, and 2016. The germplasm lines were arranged in a randomized complete block design with two replications and single-row plots in each test. The cotton seeds were hand-sown in a field covered with plastic mulch, which was applied directly by a machine in April. The plot length was 4 m, the row and plant spacings were 0.80 m and 0.26 m, respectively, and the seedlings were thinned to 16 plants plot^− 1^.

For verification of the identified QTLs, an interspecific backcross inbred line (BIL) population consisting of 176 lines was used. The BILs were produced via a cross between *G. barbadense* Hai7124 and *G. hirsutum* CRI36, using CRI36 as the recurrent parent for backcrossing with F1 to produce BC1F1, followed by eight generations of selfing. The 176 BILs and two parents were planted in three locations (Alaer, Xinjiang, 40.55°N, 81.28°E; Sanya, Hainan, 18.41°N, 109.20°E, and Anyang, Henan, 36.06°N, 114.49°E) in the 2016 growing season. The BILs and two parents were arranged in a randomized complete block design with two replications and single-row plots in each location. The planting patterns in Anyang and Sanya were the same as in the natural population, except for Alaer, Xinjiang, where a high seeding rate, with a plant spacing of 0.11 m, a row spacing of 0.38 m, a plot length of 5 m, and a total of 50 plants, was used. Crop management practices followed local recommendations for the production area.

### Phenotypic measurements and analysis

In July of each year, white flowers (or yellow flowers in the case of *G. barbadense*) present in the middle fruiting branches were tagged, and this time point was defined as 0 DPA. Six normally growing bolls, without insect or disease damage, were randomly sampled in each line in each replication at 10, 15, 20 and 25 DPA. These samples were placed on ice in the field and then stored at 4 °C for phenotype testing. To improve the measurement efficiency and accuracy of the results, only the ovules in the middle of the boll were measured for FL using the water-boiling method [28]. At plant maturity, open boll samples were harvested by hand in each test to evaluate FL using a High-Volume Instrument (HVI) 900 (Test Center of Cotton Fiber Quality affiliated with the Agriculture Ministry of China, Institute of Cotton Research, Chinese Academy of Agriculture Science, Anyang, Henan, China).

Statistical analyses, including analysis of variance (ANOVA) of FL and average growth rate (AGR) for the natural and BIL populations across environments, were performed using SPSS 23.0 (IBM, New York, USA). The combined broad-sense heritability (*H*^*2*^) of FL at 10, 15, 20, and 25 DPA and at maturity in different environments was estimated with QTL ICIMapping 4.1.0.0 [29].

### Genotyping and SNP marker filtering

Genomic DNA was extracted from fresh young leaves using a miniprep method [2]. The natural population was genotyped using the Cotton 63 K Illumina Infinium SNP array by Emei Tongde Technology Development [27]. The standards employed to control the quality of the SNP data were as follows: minor allele frequency (MAF) ≥0.05, call frequency ≥ 0.8, and nonzero genotype frequency of homozygosity. The probe sequences were extracted from the SNP array and aligned to the *G. hirsutum* L. (TM-1) reference genome [30]. The SNP markers for further association analysis were filtered as follows: (i) monomorphic or poor-quality markers were excluded and (ii) markers that could not be located to a specific physical position were eliminated. The qualified SNPs were selected for various analyses, including population structure and LD analyses. A neighbor-joining (NJ) phylogenetic tree was used to assess the genetic diversity of the 93 cotton inbred lines and cultivars. The population structure of the 83 upland cotton lines was estimated using a Bayesian Markov Chain Monte Carlo model (MCMC) with STRUCTURE 2.3.4 software. The *K* value, or the estimated number of populations, which was tested from 1 to 10, was confirmed with five repetitions of independent runs. The length of the burn-in period and number of MCMC replications after burn-in were set to 10,000 and 100,000, respectively. The TASSEL 5.0 and Power Marker 3.25 programs were employed to estimate the LD and polymorphism information content (PIC) of the SNP markers, respectively [31].

### Identification of FL QTLs based on SNPs associated with fiber length at different developmental time points

In this study, TASSEL 5.0 was used to conduct the GWAS with the Q model. The Q model was performed using a general linear model (GLM) that considered the population structure as a fixed effect [32]. The uniform Bonferroni threshold for the significance of associations between traits and SNPs was *p* < 6.51 × 10^− 5^ (*p* = 1/n, n = marker numbers, −log_10_(1/15369) ≈ 4.19), which has been widely applied in recent studies [33–35]. The R package CMplot was used to draw Manhattan plots. According to previous studies, significant SNPs within a specific LD decay distance on each chromosome were considered to belong to the same QTL [9, 32].

### Primer design for high-resolution melting (HRM) analysis in the BIL population

A high-resolution melting (HRM) analysis, which generates different curves between homozygous and heterozygous sites based on different melting temperatures of PCR products, was used for SNP detection. All PCR products with the same or different genotypes can be automatically grouped by LightCycler 480 Gene Scanning software, which has successfully been applied for the genotyping of SNPs in interspecific populations [4, 36]. The candidate SNPs obtained from the above GWAS of the natural population were first screened for homozygous polymorphisms between CRI36 and Hai7124. These screened array sequences were employed in primer design for HRM analysis of the BIL populations. Sequence-specific primers were designed using Primer-BLAST from NCBI (https://www.ncbi.nlm.nih.gov/tools/primer-blast/).

### Candidate gene mining

To obtain potential candidate genes, gene sequences within an LD region of a specific size on each chromosome, centered on the significant SNPs in the natural population, were extracted [37]. Blast2Go was used to classify the candidate genes according to biological processes, molecular functions and cellular components [38]. The NCBI non-redundant database was employed for the annotation of gene functions. To reveal the general pattern of expression of the candidate genes, we analyzed transcriptome sequencing data for cotton fiber samples in our laboratory. Ten uniformly mixed DPA fibers from two long-fiber cotton varieties (Changrongmian 601 and J02–508) and two short-fiber cotton variety (Liao 1779 and 69–6025-12) were collected and used for sequencing analysis. Moreover, transcriptome sequencing data for the dynamic developmental fibers (10, 20 and 25 DPA) of TM-1 were employed as a reference [30].

To further verify the expression trends of the candidate genes, a qRT-PCR analysis was performed. Total RNA in fibers from four developmental time-points (10, 15, 20 and 25 DPA) of two long-fiber lines (Msco-12 and EJing55173) and two short-fiber lines (JiShengNaiYan79202 and ShaCheTuMian) was extracted with the RNAPrep Pure Plant kit (Polysaccharides & Polyphenolics-rich) (TIANGEN BIOTECH CO., LTD.), respectively. Each genotype had three biological replications. Following the synthesis of cDNA using the TransScript One-Step gDNA Removal and cDNA Synthesis SuperMix (TRANSGEN BIOTECH), the TransStart Top Green qPCR SuperMix (TRANSGEN BIOTECH) was used to perform qRT-PCR according to the manufacture’s instructions. The qRT-PCR reaction conditions was set as the following in Mastercycler ep realplex S (Eppendorf, Hamburg, Germany): the first step was 94 °C (30 s) for DNA polymerase activation, the second step was 45 cycles of 94 °C (5 s), 58 °C (15 s), and 72 °C (12 s), the third step was to add a default process of melting curve analysis, and the last step was 4 °C (1 min). In this study, the Histone3 (AF024716) was used as a housekeeping gene and the relative expression level of the candidate genes was calculated with 2^-△△CT^ method [39].

## Results

### Phenotypic variations in dynamic FL among upland cotton accessions

Extensive phenotypic variation in FL at five fiber developmental time points (10, 15, 20, and 25 DPA, and maturity) was observed in this association panel of 83 upland cotton accessions tested in Anyang over three consecutive years from 2014 to 2016 (Table 1 and Additional file 2: Figure S1). The minimum FL at 10 DPA was 7.17 mm, the maximum was 18.00 mm, and the mean FLs at 10 DPA in 2014, 2015 and 2016 were 10.87, 10.80 and 14.47 mm, respectively. The FLs recorded at 15, 20, and 25 DPA and at maturity exhibited wide ranges of 10.13–28.50 mm, 20.57–34.25 mm, 17.93–37.67 mm and 22.10–34.87 mm, respectively, with means of 19.20–24.30 mm, 26.18–30.56 mm, 25.12–31.33 mm and 28.47–28.88 mm in the three years (Table 1). The mean coefficients of variation (CVs) for FL at 10, 15, 20, and 25 DPA and maturity were 11.29, 10.09, 7.56, 7.69 and 6.45%, respectively. These data indicated that the FL in the natural population showed higher variation at early developmental time points.

**Table 1:** Statistics of phenotypic variations in the dynamic fiber length and growth rates in upland cotton

Trait	Environment	Stage	Inbred lines			SD	Skewness	Kurtosis	CV (%)
			Min	Max	Mean				
Fiber length (mm)	Anyang-Yf 2014	10 DPA	7.17	14.63	10.87	1.55	0.31	0.84	14.26
		15 DPA	15.53	25.83	20.96	2.01	0.22	0.31	9.59
		20 DPA	20.57	30.27	26.18	2.57	0.52	1.29	9.80
		25 DPA	17.93	28.23	25.12	1.93	−1.54	3.19	7.68
		Maturity	23.14	33.8	28.48	1.78	0.73	5.67	6.25
	Anyang-Yf 2015	10 DPA	7.92	15.46	10.8	1.11	1.03	3.65	10.28
		15 DPA	10.13	23.42	19.2	2.41	−1.73	3.83	12.53
		20 DPA	22	31.83	28.35	1.96	−1.23	2.07	6.91
		25 DPA	19.33	34.75	30.91	2.66	−1.31	3.61	8.61
		Maturity	22.1	32.9	28.47	1.86	−0.46	1.19	6.53
	Anyang-Xt 2016	10 DPA	11	18	14.47	1.35	0.05	−0.03	9.33
		15 DPA	17	28.5	24.3	1.98	−0.73	1.5	8.15
		20 DPA	24.33	34.25	30.56	1.82	−0.34	0.78	5.96
		25 DPA	26	36.29	31.33	2.12	0.32	0.77	6.77
		Maturity	22.3	34.87	28.88	1.8	−0.25	2.42	6.23
Fiber growth rate (mm/day)	Anyang-Yf 2014	0–10 DPA	0.72	1.46	1.09	0.16	0.3	0.84	14.68
		10–15 DPA	0.57	3.41	2.02	0.54	0.06	0.77	26.73
		15–20 DPA	−0.61	2.79	1.04	0.62	0.3	0.72	59.62
		20–25 DPA	−1.55	0.56	−0.22	0.46	−0.59	− 0.13	−209.09
		25-maturity	0.04	0.32	0.13	0.06	0.82	0.88	46.15
	Anyang-Yf 2015	0–10 DPA	0.79	1.54	1.08	0.11	1.03	3.65	10.19
		10–15 DPA	0.02	2.58	1.68	0.46	−1.35	2.59	27.38
		15–20 DPA	0.16	3.7	1.83	0.58	0.57	1.81	31.69
		20–25 DPA	−0.55	1.87	0.52	0.47	0.43	0.76	90.38
		25-maturity	−0.27	0.21	−0.09	0.08	0.8	1.71	−88.89
	Anyang-Xt 2016	0–10 DPA	1.1	1.8	1.45	0.14	0.05	−0.03	9.66
		10–15 DPA	0.45	2.8	1.95	0.42	−0.8	1.19	21.54
		15–20 DPA	0.3	2.45	1.26	0.43	0.76	0.77	34.13
		20–25 DPA	−0.68	1.51	0.18	0.44	0.29	0.14	244.44
		25-maturity	−0.29	0.05	−0.1	0.06	0	0.15	−60.00

*SD* standard deviation, *CV* coefficient of variation

An ANOVA showed that the genotype, environment and genotype × environment interaction had significant effects on FL at each time point of 10, 15, 20 and 25 DPA and maturity (Table 2). A further partition of variances showed that the broad sense heritability (*H*^*2*^) values of FL at 10, 15, 20, and 25 DPA and maturity were 49.14, 47.56, 60.63, 71.06, and 92.45%, respectively (Table 2), suggesting that the *H*^*2*^ increased as the fibers elongated and grew after 10–15 DPA and that the FL at maturity was highly inherited. However, only approximately ½ of the phenotypic variation in FL was accounted for by the genetic variation at 10 and 15 DPA, indicating that the FL is more influenced by environmental factors during early developmental time-points than at later time-points.

**Table 2:** ANOVA and broad-sense heritability analysis of fiber length and growth rates at different developmental time-points

Trait	Stage	Variation source	SS	DF	MS	*F*-value	*H*^*2*^ (%)
Fiber length	10 DPA	Genotype	472.02	83	5.69	8.24^**^	49.14
		Environment	1149.72	2	574.86	833.65^**^	
		Environment × genotype	648.32	140	4.63	4.63^**^	
		Error	142.05	206	0.69		
	15 DPA	Genotype	763.17	83	9.19	7.04^**^	47.56
		Environment	2023.60	2	1011.80	775.77^**^	
		Environment × genotype	1235.36	142	8.70	6.67^**^	
		Error	267.37	205	1.30		
	20 DPA	Genotype	1076.94	83	12.98	5.69^**^	60.63
		Environment	1236.33	2	618.17	271.19^**^	
		Environment × genotype	967.14	139	6.96	3.05^**^	
		Error	476.40	209	2.28		
	25 DPA	Genotype	1591.15	83	19.17	8.48^**^	71.06
		Environment	2910.86	2	1455.43	643.91^**^	
		Environment × genotype	984.94	143	6.89	3.04^**^	
		Error	472.40	209	2.26		
	Maturity	Genotype	1179.88	83	14.22	61.10^**^	92.45
		Environment	3.44	2	1.72	7.40^**^	
		Environment × genotype	147.00	166	0.89	3.82^**^	
		Error	52.58	226	0.23		
Fiber growth rate	0–10 DPA	Genotype	4.72	83	0.06	8.24^**^	51.90
		Environment	11.50	2	5.75	833.65^**^	
		Environment × genotype	6.48	140	0.05	6.71^**^	
		Error	1.42	206	0.01		
	10–15 DPA	Genotype	34.84	83	0.42	5.39^**^	39.96
		Environment	7.94	2	3.97	51.02^**^	
		Environment × genotype	69.86	136	0.51	6.60^**^	
		Error	15.02	193	0.08		
	15–20 DPA	Genotype	51.76	83	0.62	3.98^**^	45.27
		Environment	49.10	2	24.55	156.86^**^	
		Environment × genotype	76.79	136	0.56	3.60^**^	
		Error	30.52	195	0.16		
	20–25 DPA	Genotype	38.77	83	0.47	2.37^**^	42.57
		Environment	36.59	2	18.29	93.17^**^	
		Environment × genotype	50.42	138	0.37	1.86^**^	
		Error	38.87	198	0.20		
	25-maturity	Genotype	1.05	83	0.01	2.94^**^	44.70
		Environment	4.39	2	2.19	508.93^**^	
		Environment × genotype	1.48	143	0.01	2.40^**^	
		Error	0.89	206	0.00		

### Phenotypic variations in the growth rate of fiber length

**Fig. 1:**
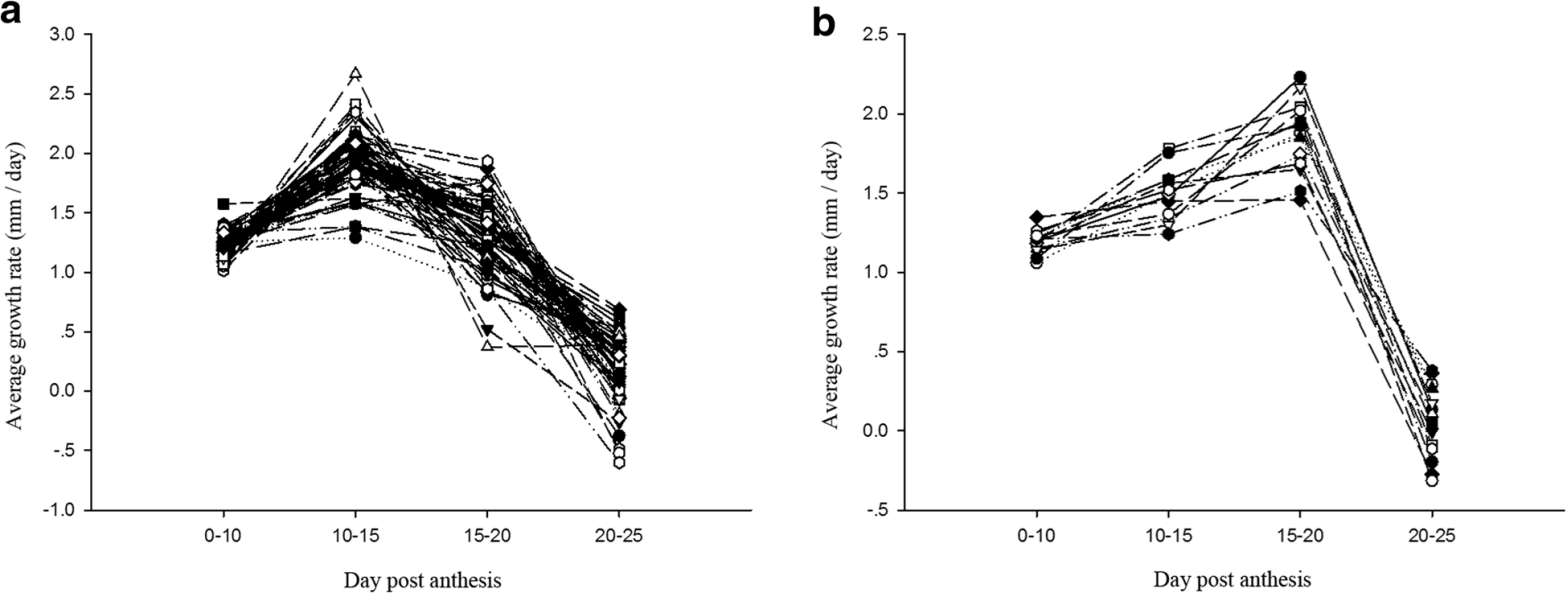
Trend of AGRs in upland cotton accessions between two adjacent periods at the fast-elongation stage. **a** The fastest fiber growth rate (AGR) occurred from 10 to 15 DPA in 69 upland cotton accessions. **b** The fastest fiber growth rate AGR occurred from 15 to 20 DPA in 14 upland cotton accessions

The growth rates of the fibers between two adjacent periods were further calculated in each of the three tests. Among the three tests, the average growth rates (AGRs) from 0 to 10 DPA, 10 to 15 DPA, 15 to 20 DPA, 20 to 25 DPA and 25 DPA to maturity were 1.21, 1.88, 1.38, 0.16 and − 0.14 mm per day, respectively. The AGR was positive from 0 to 25 DPA, and the fastest AGR was observed between 10 and 15 DPA. In contrast, dehydration of the fiber cell led to fiber shortening in maturity, which explains the observed negative AGR from 25 DPA to maturity. Based on the trend of AGRs at the fast elongation stage in the three tests, the upland cotton population can be divided into two clusters (Fig. 1). As shown in Fig. 1a (69 upland cotton accessions), the fastest AGR occurred from 10 to 15 DPA, and the AGR then gradually decreased from 15 to 20 DPA and 20 to 25 DPA. In contrast, the AGR was lower from 10 to 15 DPA than from 15 and 20 DPA, as shown in Fig. 1b (14 upland cotton accessions). This result indicated that the fastest AGR preferentially occurred from 10 to 15 DPA in most of the upland cotton accessions. Among the 14 upland cotton accessions in Fig. 1b, 10 genotypes were grouped in subpopulation 1 (P1), accounting for 21.74% in P1. In contrast, four genotypes were grouped in subpopulation 2 (P2), occupying 10.81% in P2 (Additional file 1: Table S1). This result indicated that the rate of the fastest AGR occurring from 15 to 20 DPA in P1 is higher than that in P2.

The ANOVA results indicated that the genotype, environment and genotype × environment interaction had significant effects (*P* < 0.01) on the fiber growth rate from 0 to 10 DPA, 10 to 15 DPA, 15 to 20 DPA, 20 to 25 DPA and 25 DPA to maturity (Table 2). Moreover, the *H*^*2*^ values for the fiber growth rate from 0 to 10 DPA, 10 to 15 DPA, 15 to 20 DPA, 20 to 25 DPA and 25 DPA to maturity were 51.90, 39.96, 45.27, 42.57, and 44.70%, respectively (Table 2), suggesting that the fiber growth rate was easily influenced by the environment in these time-points.

### LD and grouping of upland cotton germplasm lines based on 63 K SNP array analysis

**Fig. 2:**
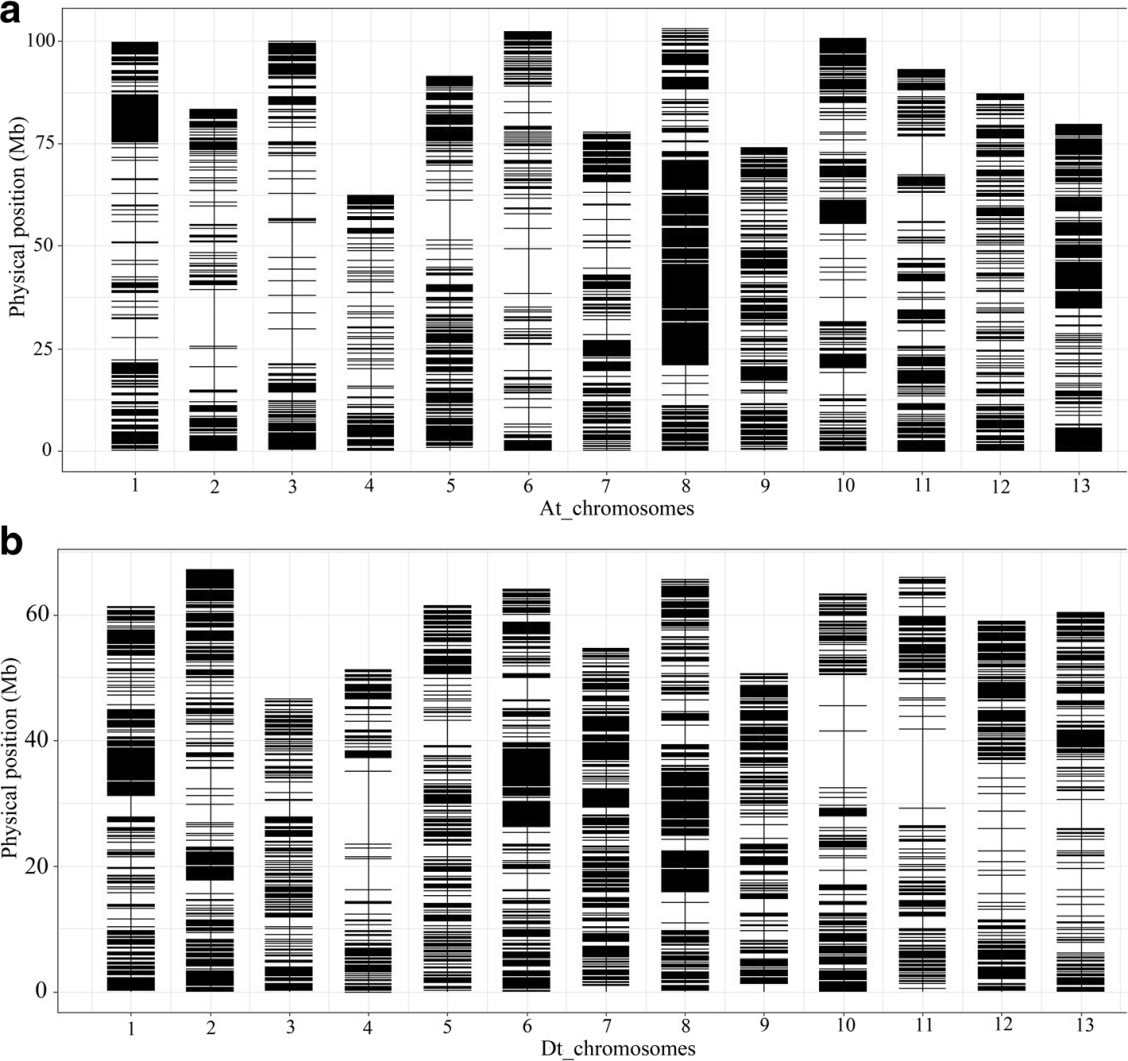
Genome-wide single-nucleotide polymorphism (SNP) density in the entire association mapping panel. **a** SNP distribution on the 13 chromosomes of the A subgenome. **b** SNP distribution on the 13 chromosomes of the D subgenome. The dark and white horizontal bars indicate genomic regions that are rich and poor in SNPs, respectively

The Cotton 63 K Illumina Infinium SNP array contained 63,058 SNPs, and 31,765 of these SNPs were monomorphic or poor-quality markers. Among the remaining SNPs, 15,924 SNPs showed BLAST matches to two or more locations in the reference genome of TM-1 and were therefore excluded. Finally, 15,369 SNPs exhibited unique positions and were therefore used for population structure assessment and LD analysis. These SNPs were not evenly distributed on each chromosome, and the marker density varied across the whole cotton genome. The lowest SNP marker density was one SNP per 324.43 kb on chromosome At6, whereas chromosome Dt8 showed the highest marker density, equal to one SNP per 67.93 kb (Fig. 2 and Table 3). The mean PIC values of the A and D subgenomes were 0.3732 and 0.3982, respectively. Among all chromosomes, the PIC values ranged from 0.3510 to 0.4079 (Table 3).

**Table 3:** Summary the SNPs mapped on each chromosome and the LD decay estimated for each chromosome

Chromosome	Number of SNPs	Chr. length (Mb)	Density of SNP (kb/SNP)	PIC	LD (Mb)
At1	686	99.88	145.60	0.3510	2.00
At2	309	83.45	270.06	0.3785	0.30
At3	417	100.36	240.67	0.3673	0.30
At4	223	62.91	282.11	0.3695	0.30
At5	705	92.05	130.57	0.3794	0.60
At6	318	103.17	324.43	0.3794	0.40
At7	514	78.25	152.24	0.3720	0.60
At8	1305	103.63	79.41	0.3710	3.70
At9	526	75.00	142.59	0.3806	1.00
At10	640	100.78	157.47	0.3727	0.80
At11	542	93.32	172.18	0.3801	0.40
At12	488	87.48	179.26	0.3795	0.30
At13	870	79.96	91.91	0.3701	2.10
Dt1	769	61.46	79.92	0.3869	2.30
Dt2	865	67.28	77.78	0.3843	0.60
Dt3	372	46.69	125.51	0.4016	0.20
Dt4	295	51.45	174.41	0.4066	0.20
Dt5	575	61.93	107.70	0.4077	0.30
Dt6	751	64.29	85.61	0.3867	1.50
Dt7	761	55.31	72.68	0.4079	0.60
Dt8	970	65.89	67.93	0.3815	2.70
Dt9	576	51.00	88.54	0.3976	0.40
Dt10	520	63.37	121.87	0.3958	0.50
Dt11	397	66.09	166.47	0.4072	0.50
Dt12	537	59.11	110.07	0.4075	0.30
Dt13	438	60.53	138.20	0.4053	0.50

*PIC* polymorphism information content

LD decay is the physical distance in the genome at which the value of r^2^ is half of the maximum value

**Fig. 3:**
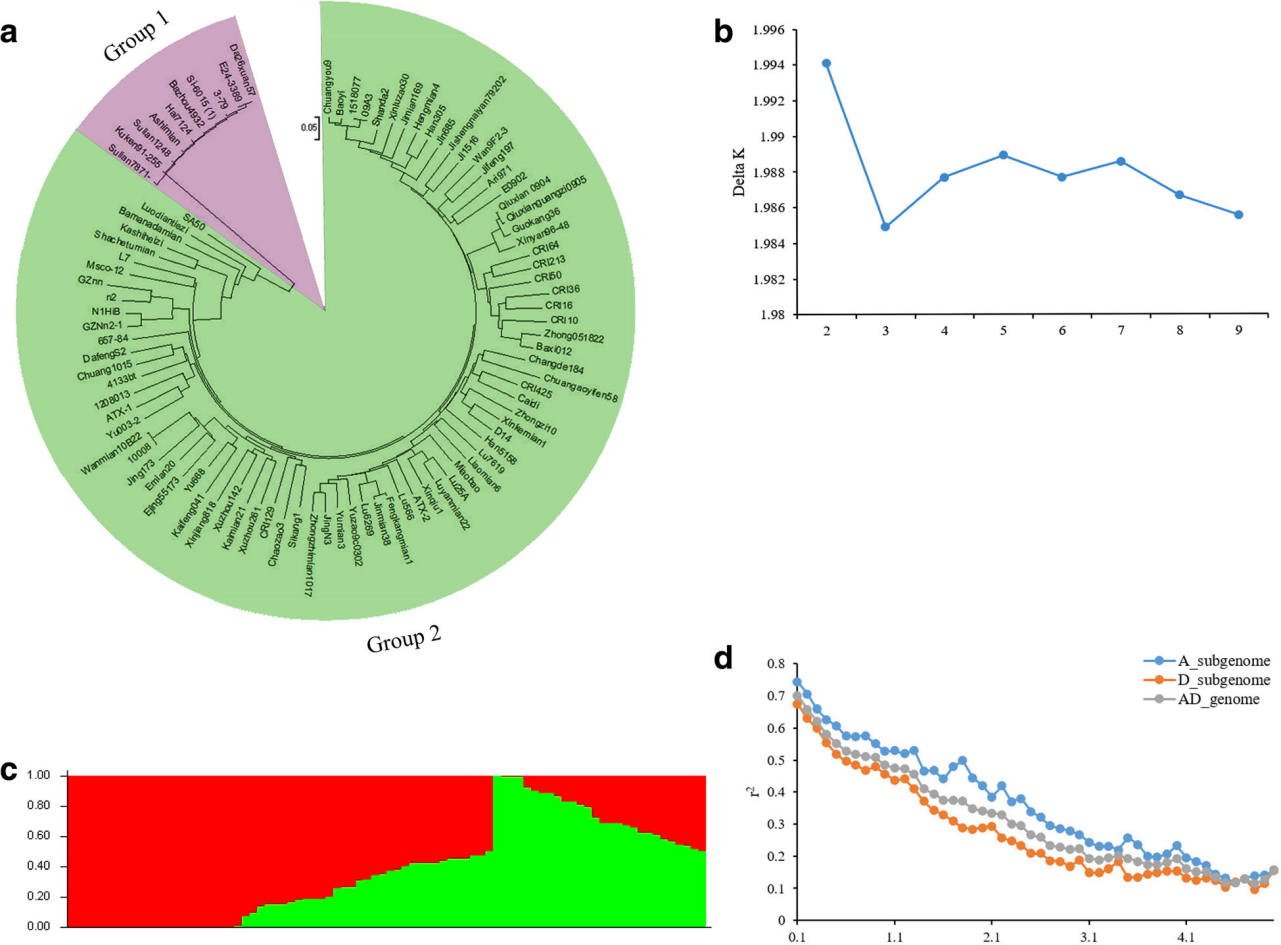
Population structure and linkage disequilibrium (LD) decay of upland cotton accessions. **a** NJ phylogenetic analysis of 93 cotton accessions; purple and green represent Groups 1 and 2, respectively. **b** Population structure of 83 upland cotton accessions based on a STRUCTURE analysis with k = 2; red and green represent subpopulation 1 (P1) and subpopulation 2 (P2), respectively (**c**) ΔK based on the rate of change in LnP (K) between successive K values. **d** LD decay determined based on squared correlations of allele frequencies (r^2^) against the distance between polymorphic sites in the A subgenome (blue), D subgenome (orange) and AD genome (gray)

The r^2^ statistic (the squared Pearson correlation coefficient) was employed to calculate LD. The LD values of the A subgenome, D subgenome and AD genome were 2.1, 1.6 and 1.9 Mb, respectively, and the value of r^2^ was half of the maximum value (Fig. 3d). The LD value obtained for the A subgenome was higher than those found for the D subgenome and the AD genome. The LD decay varied from 0.2 to 3.7 Mb depending on the chromosome of upland cotton; however, the LD decay on chromosome Dt8 was 2.7 Mb, which was higher than that on other chromosomes of the D subgenome (Table 3). These results indicated significant differences in LD decay between subgenomes and chromosomes.

The 93 cotton accessions were divided into two species groups through a NJ phylogenetic analysis, with Group 1 containing all 10 *G. barbadense* lines and Group 2 consisting of all 83 upland accessions, as expected (Fig. 3a). This result verified the reliability of the genotyping of the cotton accessions in this study. Using a Bayesian Markov Chain Monte Carlo model (MCMC) with STRUCTURE 2.3.4 software, the 83 upland cotton accessions were further grouped into two subpopulations (Fig. 3c and Additional file 1: Table S1), consistent with the results of a previous study [10].

### Association analysis between SNPs and FL at different developmental time points

We subsequently performed an association analysis for the 83 upland cotton lines using the GLM (Q) model. At 10, 15, 20 and 25 DPA and maturity, a total of 25, 38, 57, 89 and 88 SNPs, respectively, showed significant correlations with the FL tested in the three tests (Additional file 3: Figure S2). Through a further analysis, 60 common SNPs were detected in at least two tests and two FL developmental time points (Table 4). These significant SNPs were distributed on 13 chromosomes: At8, At9, At12, Dt2, Dt4, Dt5, Dt7, Dt8, Dt9, Dt10, Dt11, Dt12 and Dt13. Among these 13 chromosomes, Dt4 exhibited the maximum number of 10 SNPs. Most importantly, two SNPs, i60962Gt and i11417Gh, were associated with the FL at four different time-points (Table 4). Among the 60 common SNPs, two and four SNPs were significantly associated with FL at 10 and 15 DPA, respectively, whereas most of the SNPs were found to be associated with FL at 20 or 25 DPA and maturity (Table 4). Moreover, i56109Gb on Dt5 and i36390Gh on Dt9 were associated with FL at 15, 20 and 25 DPA and at 10, 15 and 25 DPA, respectively (Table 4). Considering the specific LD decay distance on each chromosome as the QTL confidence interval, these 60 significant SNPs formed 21 QTL regions, each of which explained 11.06–60.01% of the phenotypic variation in FL (Table 4).

**Table 4:** Summary of SNPs significantly associated with FL traits at different developmental time-points

QTL	SNP	Chr.	Site	-log10P	R^2^ (%)	Traits	Reported previously
FL-QTL-1	i43923Gh	At8	6,889,977	4.70–7.91	20.54–43.50	25 DPA^a,b^, Maturity^b,c^	[16, 22]
	i47458Gh	At8	6,926,743	4.64–7.77	20.20–42.63	25 DPA^a,b^, Maturity^b,c^	
	i46694Gh	At8	6,947,649	4.64–7.77	20.20–42.63	25 DPA^a,b^, Maturity^b,c^	
FL-QTL-2	i55088Gb	At9	60,651,133	4.27–12.61	11.71–49.44	25 DPA^a,b^, Maturity^a,b^	
FL-QTL-3	i06403Gh	At9	70,095,261	5.40–6.94	14.56–20.77	20 DPA^b^, 25 DPA^a,b^, Maturity^a,b,c^	
	i06405Gh	At9	70,132,917	5.40–6.94	14.56–20.77	20 DPA^b^, 25 DPA^a,b^, Maturity^a,b,c^	
FL-QTL-4	i07778Gh	At12	562,289	4.21–5.97	18.57–27.78	25 DPA^a,b^, Maturity^b,c^	
FL-QTL-5	i16326Gh	At12	62,237,916	4.20–9.15	17.91–53.60	20 DPA^b,c^, 25 DPA^a^, Maturity^b,c^	[20]
	i31671Gh	At12	62,374,407	4.21–8.99	18.03–52.56	20 DPA^b,c^, 25 DPA^a^, Maturity^b,c^	
	i34476Gh	At12	62,421,193	4.20–9.15	17.91–53.60	20 DPA^b,c^, 25 DPA^a^, Maturity^b,c^	
	i36493Gh	At12	62,584,169	4.20–9.15	17.91–53.60	20 DPA^b,c^, 25 DPA^a^, Maturity^b,c^	
	i63008Gt	At12	62,631,642	4.21–8.99	18.03–52.56	20 DPA^b,c^, 25 DPA^a^, Maturity^b,c^	
	i08069Gh	At12	62,823,942	4.20–9.15	17.91–53.60	20 DPA^b,c^, 25 DPA^a^, Maturity^b,c^	
	i46470Gh	At12	62,896,861	4.22–8.99	18.03–52.56	20 DPA^b,c^, 25 DPA^a^, Maturity^b,c^	
FL-QTL-6	i05769Gh	Dt2	67,113,037	5.38–8.75	14.59–28.90	15 DPA^c^, 25 DPA^a,b^, Maturity^b,c^	
FL-QTL-7	i60907Gt	Dt4	196,659	4.24–9.87	18.05–59.97	20 DPA^b^, 25 DPA^a,b^, Maturity^b,c^	
	i47681Gh	Dt4	200,376	4.24–9.87	18.05–59.97	20 DPA^b^, 25 DPA^a,b^, Maturity^b,c^	
FL-QTL-8	i27361Gh	Dt4	3,262,973	4.23–5.36	11.10–14.30	20 DPA^b,c^, 25 DPA^a,b^, Maturity^b^	
	i39155Gh	Dt4	3,280,556	4.23–5.36	11.10–14.30	20 DPA^b,c^, 25 DPA^a,b^, Maturity^b^	
	i36124Gh	Dt4	3,387,942	4.29–5.66	18.30–25.97	20 DPA^b,c^, 25 DPA^a,b^, Maturity^b,c^	
	i20432Gh	Dt4	3,496,432	4.45–6.57	19.22–31.58	20 DPA^b^, 25 DPA^a,b^, Maturity^b,c^	
	i17744Gh	Dt4	3,544,396	4.31–6.61	18.41–31.78	20 DPA^b^, 25 DPA^a,b^, Maturity^b,c^	
	i12503Gh	Dt4	3,551,693	4.29–5.66	18.30–25.97	20 DPA^b,c^, 25 DPA^a,b^, Maturity^b,c^	
	i24940Gh	Dt4	3,731,918	4.37–6.16	18.73–28.94	20 DPA^b^, 25 DPA^a,b^, Maturity^b,c^	
	i45150Gh	Dt4	3,735,149	4.37–6.16	18.73–28.94	20 DPA^b^, 25 DPA^a,b^, Maturity^b,c^	
FL-QTL-9	i56109Gb	Dt5	10,061,254	4.30–6.67	18.41–32.06	15 DPA^c^, 20 DPA^b^, 25 DPA^a,b^	
FL-QTL-10	i01791Gh	Dt7	20,011,717	5.08–9.01	13.59–30.13	20 DPA^b^, 25 DPA^a,b^, Maturity^b,c^	
	i01793Gh	Dt7	20,012,463	5.36–9.85	24.23–59.73	25 DPA^a,b^, Maturity^b,c^	
FL-QTL-11	i25898Gh	Dt8	60,138,274	4.37–8.60	12.02–28.24	25 DPA^a,b^, Maturity^a,b^	[16]
	i39699Gh	Dt8	60,145,594	4.37–8.60	12.02–28.24	25 DPA^a,b^, Maturity^a,b^	
	i04589Gh	Dt8	60,209,167	4.37–8.60	12.02–28.24	25 DPA^a,b^, Maturity^a,b^	
	i52348Gb	Dt8	60,213,494	4.37–8.60	12.02–28.24	25 DPA^a,b^, Maturity^a,b^	
	i40219Gh	Dt8	60,283,929	4.37–8.60	12.02–28.24	25 DPA^a,b^, Maturity^a,b^	
	i25899Gh	Dt8	60,289,122	4.37–8.60	12.02–28.24	25 DPA^a,b^, Maturity^a,b^	
	i04592Gh	Dt8	60,327,275	4.37–8.60	12.02–28.24	25 DPA^a,b^, Maturity^a,b^	
	i47255Gh	Dt8	60,364,319	4.37–8.60	12.02–28.24	25 DPA^a,b^, Maturity^a,b^	
FL-QTL-12	i36390Gh	Dt9	35,827,578	4.50–4.53	19.30–25.02	10 DPA^b^, 15 DPA^c^, 25 DPA^a,b^	
FL-QTL-13	i06490Gh	Dt9	47,021,366	4.19–7.72	11.44–24.40	25 DPA^a,b^, Maturity^a,b^	
	i06491Gh	Dt9	47,021,806	4.19–7.72	11.44–24.40	25 DPA^a,b^, Maturity^a,b^	
FL-QTL-14	i20045Gh	Dt10	13,951,286	4.59–12.82	19.89–60.01	25 DPA^a,b^, Maturity^a,b,c^	[20]
	i39524Gh	Dt10	13,996,422	4.59–12.82	19.89–60.01	25 DPA^a,b^, Maturity^a,b,c^	
	i35125Gh	Dt10	13,999,766	4.59–12.82	19.89–60.01	25 DPA^a,b^, Maturity^a,b,c^	
	i38078Gh	Dt10	14,128,474	4.59–12.82	19.89–60.01	25 DPA^a,b^, Maturity^a,b,c^	
	i37666Gh	Dt10	14,155,678	4.59–12.82	19.89–60.01	25 DPA^a,b^, Maturity^a,b,c^	
	i39425Gh	Dt10	14,181,220	4.59–12.82	19.89–60.01	25 DPA^a,b^, Maturity^a,b,c^	
	i27558Gh	Dt10	14,295,765	4.59–12.82	19.89–60.01	25 DPA^a,b^, Maturity^a,b,c^	
FL-QTL-15	i14259Gh	Dt10	55,580,911	4.86–6.62	22.11–33.94	25 DPA^a,b^, Maturity^a,b^	[9]
	i20114Gh	Dt10	55,589,792	4.86–6.62	22.11–33.94	25 DPA^a,b^, Maturity^a,b^	
FL-QTL-16	i60963Gt	Dt11	8,678,122	4.59–12.82	19.89–60.01	25 DPA^a,b^, Maturity^a,b,c^	
FL-QTL-17	i07222Gh	Dt11	16,662,698	5.14–12.16	13.74–46.69	10 DPA^b^, 25 DPA^a,b^, Maturity^a,b,c^	
FL-QTL-18	i60962Gt	Dt11	24,030,081	4.67–6.29	20.36–31.63	25 DPA^a,b^, Maturity^b,c^	[9, 10]
	i16034Gh	Dt11	24,059,057	4.67–6.29	20.36–31.63	25 DPA^a,b^, Maturity^b,c^	
	i16035Gh	Dt11	24,059,152	4.67–6.29	20.36–31.63	25 DPA^a,b^, Maturity^b,c^	
FL-QTL-19	i32896Gh	Dt12	7,215,751	4.53–9.15	19.56–53.61	25 DPA^a,b^, Maturity^a,b,c^	
	i07931Gh	Dt12	7,261,923	4.53–9.15	19.56–53.61	25 DPA^a,b^, Maturity^a,b,c^	
	i34663Gh	Dt12	7,263,333	4.53–9.15	19.56–53.61	25 DPA^a,b^, Maturity^a,b,c^	
	i34662Gh	Dt12	7,378,095	4.53–9.15	19.56–53.61	25 DPA^a,b^, Maturity^a,b,c^	
FL-QTL-20	i19464Gh	Dt12	46,546,520	4.23–7.99	17.96–44.08	25 DPA^a,b^, Maturity^a,b,c^	
FL-QTL-21	i11417Gh	Dt13	54,844,478	4.68–6.79	12.43–19.07	15 DPA^c^, 25 DPA^b^, Maturity^a,b^	[16]
	i33230Gh	Dt13	56,126,379	4.25–7.09	11.06–20.16	25 DPA^b,c^, Maturity^a,b,c^	
AGR-QTL-1	i06955Gh	At11	6,536,455	4.27	19.45	10–15 DPA^c^	
	i06960Gh	At11	6,631,727	4.19	18.83	10–15 DPA^c^	
AGR-QTL-2	i02910Gh	Dt1	58,316,772	5.02	22.61	10–15 DPA^c^, 15–20 DPA^c^	
AGR-QTL-3	i36135Gh	Dt4	7,617,473	4.45	20.02	10–15 DPA^c^	
	i33788Gh	Dt4	7,718,785	4.45	20.02	10–15 DPA^c^	
AGR-QTL-4	i08049Gh	Dt12	37,886,196	4.58	25.31	15–20 DPA^a^	
	i08062Gh	Dt12	38,398,622	4.46	28.99	15–20 DPA^a^	
	i08063Gh	Dt12	38,401,647	4.85	26.76	15–20 DPA^a^	
AGR-QTL-5	i11425Gh	Dt13	54,754,208	5.99	19.2	20–25 DPA^b^	[16]
	i11417Gh	Dt13	54,844,478	5.66	29.01	10–15 DPA^c^	

a, b and c indicate the 2014, 2015, and 2016 environments in Anyang, respectively

r^2^ is the percentage of phenotypic variance explained by the SNP

Traits indicate the FL and AGR at different developmental time-point

### Identified QTLs for the FL growth rate

**Fig. 4:**
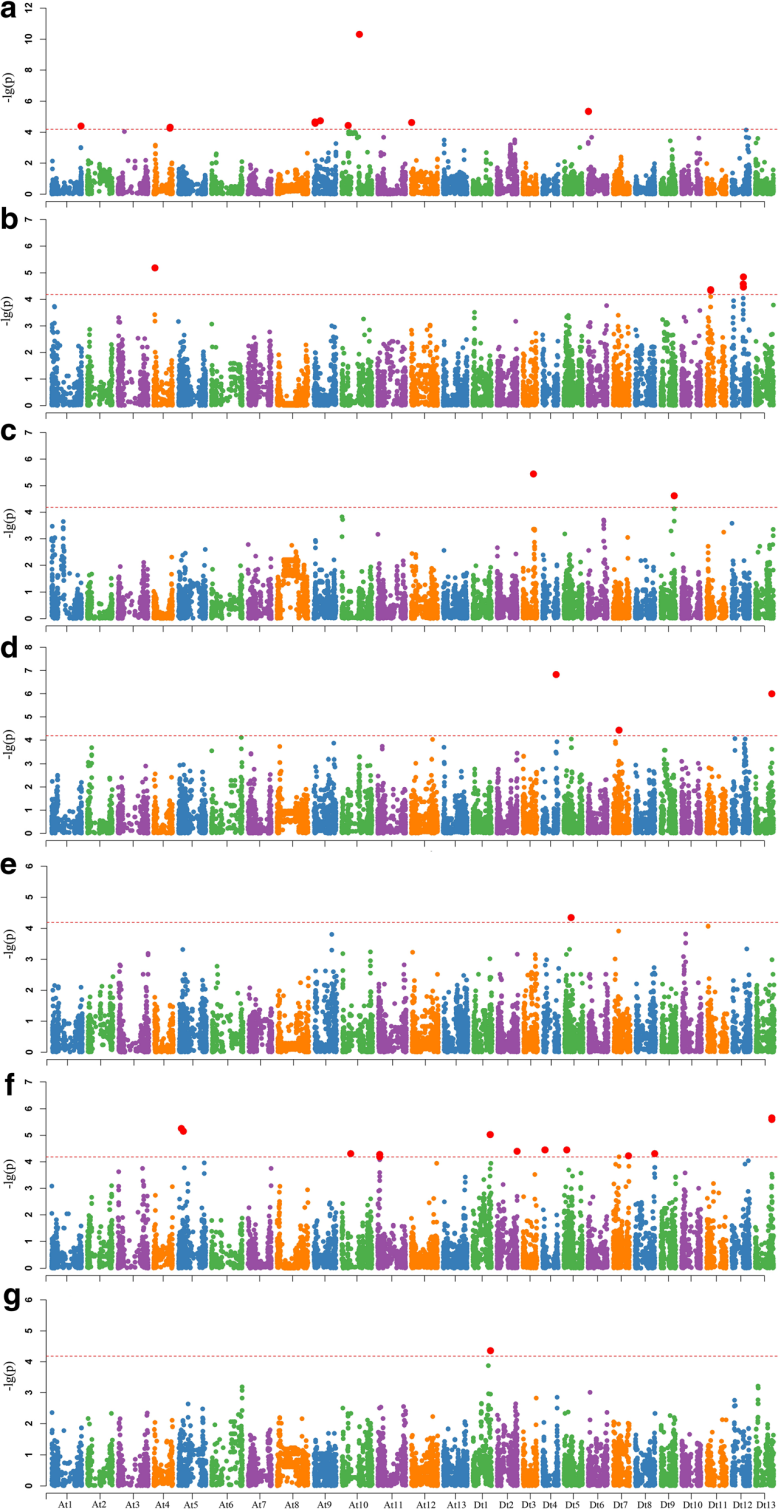
Genome-wide association study (GWAS) of the fiber length growth rate at the fast-elongation stage. The lowercase letters a and b represent Manhattan plots of the GLM for 10 to 15 DPA and 15 to 20 DPA in 2014, respectively; the letters c and d represent Manhattan plots of the GLM for 10 to 15 DPA and 20 to 25 DPA in 2015, respectively; and e, f and g represent Manhattan plots of the GLM for 0 to 10 DPA, 10 to 15 DPA and 15 to 20 DPA in 2016, respectively

The growth rate of fibers between two adjacent periods in the fast-elongation stage is an important agricultural trait that influences the final FL. Nineteen, 20, 10 and three SNPs showed significant correlations with the AGR tested in the three environments from 0 to 10 DPA, 10 to 15 DPA, 15 to 20 DPA and 20 to 25 DPA, respectively (Fig. 4). Only one common SNP, i02910Gh, could be detected from 10 to 15 DPA and from 15 to 20 DPA in 2016, but nine additional SNPs detected only in one environment and one time-point were clustered into four AGR-QTLs (Table 4). In total, 10 reliable significant SNPs formed five QTL regions, each explaining 18.83–29.20% of the phenotypic variation for AGR (Table 4). Compared between the FL-QTL and AGR-QTL, one common QTL region was located at 54.48 Mb on chromosome Dt13 (Table 4).

### Reverification of candidate SNPs in a BIL population

**Fig. 5:**
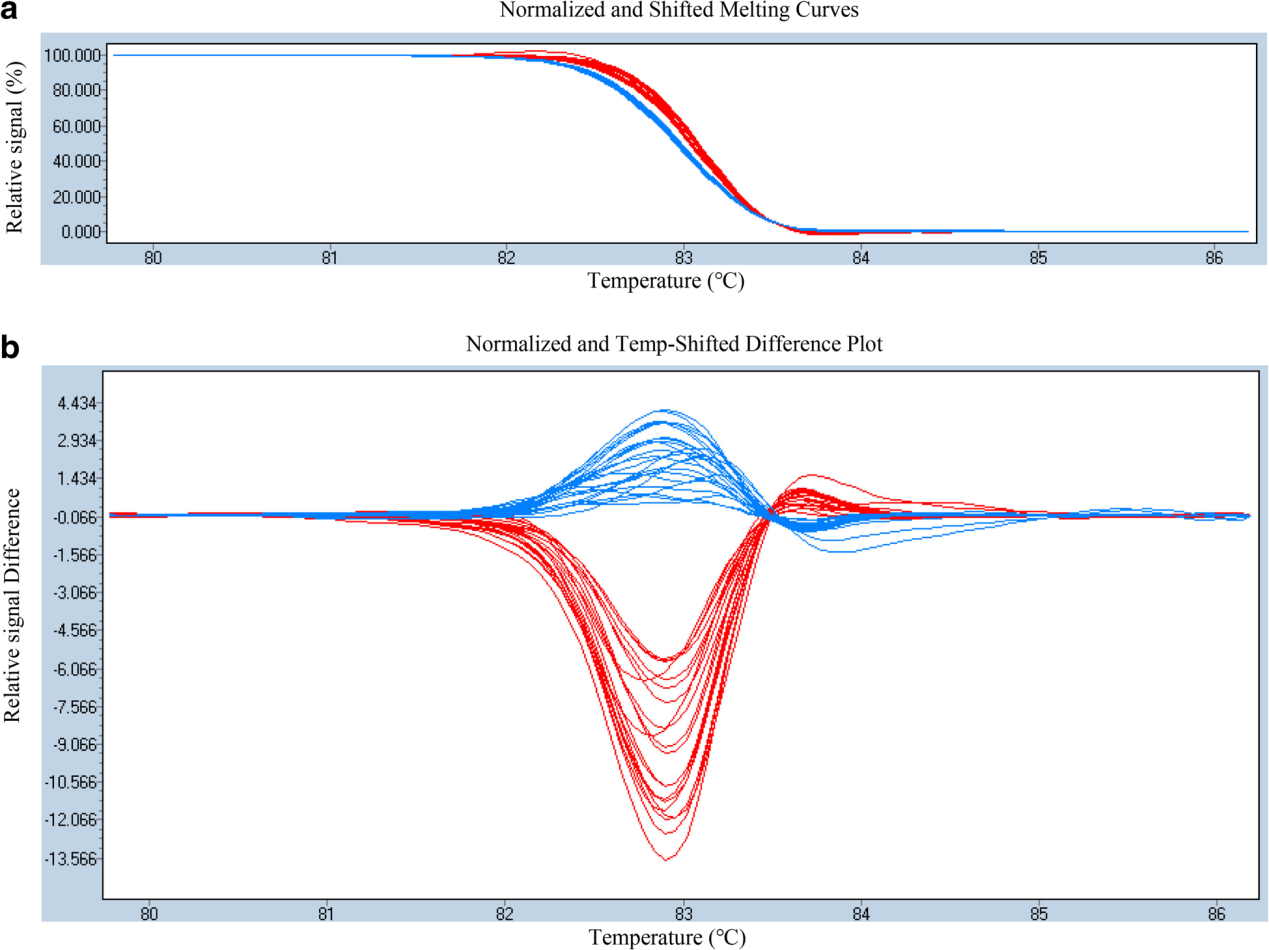
HRM analysis for validating the presence of single-nucleotide polymorphisms in BILs. **a** Original melting curves. **b** Melting curves after logarithmic calculations; the red and blue curves correspond to the CRI36 and Hai7124 genotypes, respectively

Through a NJ phylogenetic analysis, *G. barbadense* Hai7124 and upland cotton CRI36 were clustered into Group 1 and Group 2, respectively, as expected for two different tetraploid species (Fig. 3a). The FL of Hai7124 is significantly longer than that in CRI36, also as expected (Additional file 4: Table S3). Therefore, this BIL population is suitable to study the FL trait. To confirm some of the results from the above analysis, a BIL population of 176 lines and the two parents were sown at three locations in 2016, and phenotypic data for FL at maturity were obtained (Additional file 4: Table S3 and Additional file 5: Figure S3). Among the 60 candidate SNPs, seven SNPs exhibited homozygous polymorphism between CRI36 and Hai7124, and sequence-specific primers were designed to confirm these candidate SNPs in the BIL population (Additional file 6: Table S4). The candidate SNP i20432Gh was identified, and this SNP was significantly correlated with FL at maturity in the BIL population in two environments (Additional file 7: Table S5). Figure 5 shows a difference plot from an HRM analysis that discriminate different genotypes of i20432Gh in the BIL population.

### Gene ontology analysis of candidate genes associated with SNPs

We identified potential candidate genes near 60 significant SNP loci in the *G. hirsutum* TM-1 genome [30]. Based on the LD decay on each chromosome, a total of 1221 predicted genes were identified near the significant SNPs. A Gene Ontology (GO) analysis indicated that the largest number of genes was involved in intracellular and ion-binding reactions (Additional file 8: Figure S4). We also conducted a KEGG pathway enrichment analysis of all candidate genes and found that 152 genes were enriched in 65 pathways (Additional file 9: Table S6). The top three pathways containing 31 genes were involved in the biosynthesis of antibiotics and the cysteine/methionine and purine metabolism pathways. The sucrose and fatty acid metabolism pathway is known to be related to fiber development [5, 39–42]. Several genes were identified, including Gh_D11G1583 encoding cellulose synthase H1, Gh_D13G2037 encoding sucrose synthase, Gh_A09G1662 encoding glucan endo-1,3-beta-glucosidase, and Gh_A09G1061 encoding chloroplastic phospholipase A1 (Additional file 9: Table S6). The expression levels of these genes were further analyzed in the transcriptome data, as described below.

### Transcriptome and qRT-PCR analysis for mining candidate genes

**Fig. 6:**
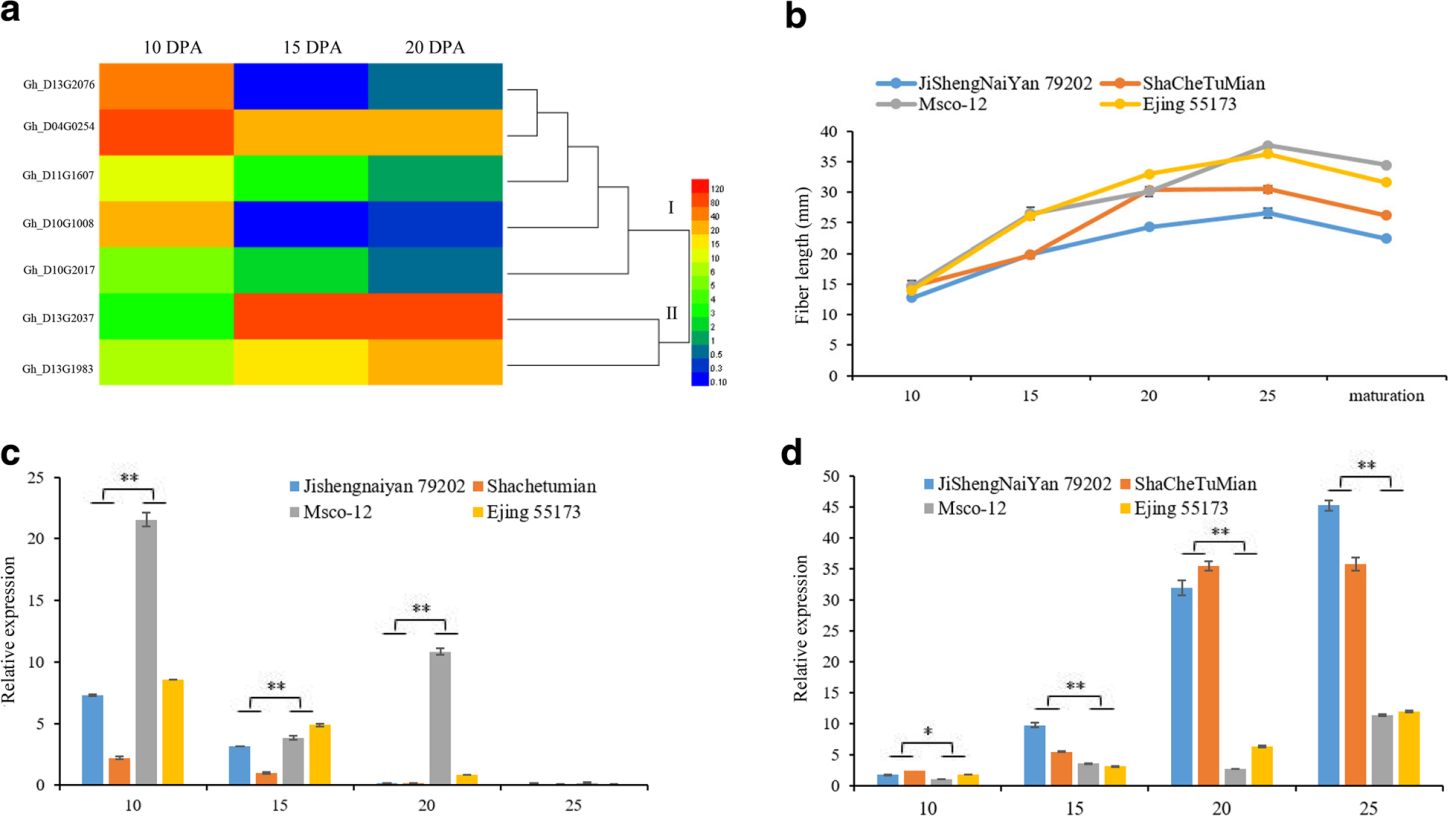
Expression of D10G1008 and D13G2037 in long- and short-fiber cultivars of upland cotton. **a** Transcript profiles of promising genes for TM-1 fibers at 10, 15 and 20 DPA. **b** Phenotypic effect values of the dynamic fiber length in two long-fiber and two short-fiber cotton varieties. **c** Expression levels of D10G1008 at four developmental stages of fiber. **d** Expression levels of D13G2037 at four developmental stages of fiber. * and ** indicate significant differences at *p* = 0.05 and 0.01, respectively

We further analyzed these candidate genes using transcriptome sequencing data generated in our laboratory (Accession ID: PRJNA400837). Among the 1221 candidate genes, 47 genes showed significant differences in expression at 10 DPA between the long- and short-fiber cotton genotypes (Additional file 10: Figure S5). These 47 genes were analyzed using transcriptome sequencing data from fibers of upland cotton TM-1 at 10, 20 and 25 DPA. Based on the changes in the expression of the genes in developing fibers, seven genes were identified (Fig. 6a). Among the seven candidate genes, the expression levels of five genes gradually decreased with fiber development (group Ι), whereas the expression levels of the two other genes gradually increased (group П). qRT-PCR was further performed to examine the expression levels of the seven genes in developing fibers (10, 15, 20 and 25 DPA) in two long-fiber genotypes and two short-fiber genotypes (Fig. 6b). Only two genes (D13G2037 and D10G1008) showed significant differential expressions. Amplified by primers qRT-D13G2037-F: 5′- ATCAAGTCCGTGCCTTGGAG -3′ and qRT-D13G2037-R: 5′- GTTGACCGCAAGTTGTTCCC -3′ with the reaction efficiency of 1.03 in the fiber samples, D13G2037 exhibited a high expression level in the developing fibers of the four upland cotton genotypes. However, but its expression level was significantly lower in the two long-fiber lines (Msco-12 and EJing55173) than in the two short-fiber lines (JiShengNaiYan79202 and ShaCheTuMian) (Fig. 6d). The reverse was true for the expression of D10G1008 (Fig. 6c), amplified by the qRT-PCR primers of D10G1008 were qRT-D10G1008-F: 5′- GTTGGGTGCTGAAGAGGTGA -3′ and qRT-D10G1008-R: 5′- TGGCCACTGGGAAGAATGTC -3′ with the reaction efficiency of 0.94 in the fiber samples. However, the expression trends of the other genes did not show significant differences between the long- and short-fiber genotypes. These data indicate that D13G2037 and D10G1008 are likely candidate genes involved in fiber elongation in upland cotton.

## Discussion

### ANOVA and inheritance analysis of the FL trait in upland cotton

The FL trait is controlled by the genotype, environment and their interactions [9, 10]. However, no information is available on FL during fiber development. In the present study, we first showed that FL at each time point of 10, 15, 20 and 25 DPA and maturity were also significantly affected by genotype (G), environment (E) and G × E (Table 2). Moreover, the heritability estimates (*H*^*2*^) for FL at 10, 15, 20 and 25 DPA and maturity were 49.14, 47.56, 60.63, 71.06 and 92.45%, respectively (Table 2). This result indicates that as the fiber developed, the trend of *H*^*2*^ showed a marked increase. In previous studies, the *H*^*2*^ values of mature FL varied in different populations, such 71.3% in a 550-RIL population [43], 85.0% in 98-RIL population [44], and 92.0% in a 503 upland cotton accessions population [9]. The result from the present study is consistent with the GWAS containing 503 upland cotton accessions.

### QTLs for the dynamic FL trait in upland cotton

According to developmental genetics, the final phenotypic traits of plants are always controlled by the dynamic expression of different QTLs during growth. The phenotypic traits of a plant at maturity, rather than the phenotypic traits at intermediate developmental stages, are commonly used to detect correlated QTLs, and therefore, some of the associated QTLs are missed. The phenotyping data obtained for 83 upland cotton accessions in various environments revealed relatively abundant variation in the FL trait during development, which showed high diversity in this natural population (Table 1 and Additional file 2: Figure S1). In this study, we first performed a GWAS of the developing FL trait in natural accessions of upland cotton based on the Cotton 63 K SNP array. A total of 88 SNPs for FL were identified in mature stages, whereas 25, 38, 57 and 89 SNPs were identified at 10, 15, 20 and 25 DPA, respectively, among the three examined environments (Additional file 3: Figure S2). Some SNPs verified at four dynamic developmental time-points were not detected in the mature stage. Similar results have previously been found for the dynamic developmental behavior of plant height and boll number in upland cotton [24, 45]. To determine the most reliable QTLs for FL, we defined QTLs detected in different studies at close or identical physical positions as belonging to the same QTL. A total of 21 QTLs (60 SNPs) underlying fiber development were inferred, and each of these might play important roles at multiple developmental time-points of fiber growth (Table 4). However, among the 21 QTLs, only two and four QTLs were found to be associated with FL at 10 and 15 DPA, respectively. Most of the QTLs were detected at 20 and 25 DPA and at maturity (Table 4). In addition, two SNPs, i56109Gb in FL-QTL-9 and i36390Gh in FL-QTL-12, were identified across four dynamic developmental time-points, not including maturity. This result indicates that examining the continual developmental of FL traits contributed to the identification of more QTLs. *H*^*2*^ was less than 50% at 10 and 15 DPA, indicating that fiber growth is susceptible to the environment at these two time-points, which likely explains why fewer SNPs were detected at 10 and 15 DPA.

### Identification and reverification of SNP loci in BILs and previous studies

There is an increasing trend of researchers jointly using multi-genetic background populations to verify the reliability of GWAS results [24, 46–48]. For example, a natural association panel and two nested association mapping (NAM) populations were employed for a GWAS of flowering time in maize, and the relevant SNPs indicated high validity for the verification of flowering time [48]. To analyze the boll number per plant (BNP) in cotton, two corresponding BILs and two recombinant inbred lines (RILs) were used for genetic analysis, and 48 potential QTLs for BNP were detected [24]. In the present study, a natural population was employed for a GWAS of the dynamic FL trait in cotton. Among the 60 significantly associated SNP loci identified in the GWAS analysis, seven SNPs showed polymorphism in the parents of the BILs. To further verify the reliability of the significantly associated SNP loci, 176 BILs were used for HRM analysis. The re-verified SNP i20432Gh was significantly correlated with FL at maturity (Additional file 7: Table S5). In the current study, significant correlations of the i60962Gt SNP with FL at maturity were verified in 503 and 719 diverse upland cotton accessions (Table 4). qFL20.1, which is located just 0.50 Mb from i20045Gh, showed a significant correlation with FL at maturity in 180 recombinant inbred lines [20]. Moreover, the SNPs identified in FL-QTL-1, FL-QTL-5, FL-QTL-11, FL-QTL-14 and FL-QTL-20 were located in hotspots for FL traits reported in previous studies (Table 4). These data suggest that the results of our GWAS for developing fibers are reliable.

### Mining candidate genes for improving FL in upland cotton

A combined analysis of KEGG pathways and transcriptome sequencing data revealed a number of candidate genes for fiber elongation (Fig. 6a and Additional file 9: Table S6). The expression in developing cotton fibers of two of these genes, D10G1008 and D13G2037, differed significantly between two long-fiber cotton varieties and two short-fiber cotton varieties. D10G1008 on Dt10 is homologous to AT4G39490 (alkane hydroxylase MAH1-like). Alkane hydroxylase MAH1-like is a member of the cytochrome P450 family and plays a key role in the biosynthesis of secondary alcohols or ketones. The overexpression of MAH1 leads to an increase in the biosynthesis of very-long-chain fatty acids (VLCFAs, C24:0 and C30:0) in Arabidopsis [49], and saturated VLCFAs promote cotton fiber cell elongation [41]. Notably, the expression of D10G1008 was significantly higher in the long-fiber varieties Msco-12 and EJing55173 than in the short-fiber varieties JiShengNaiYan79202 and ShaCheTuMian (Fig. 6c). This result supports the possibility that D10G1008 promotes fiber elongation by increasing the level of VLCFAs. The D13G2037 gene, located simultaneously in the FL-qtl-21 and AGR-qtl-5 confidence intervals, encodes sucrose synthase 4 (Susy4) in Arabidopsis. Cotton fiber mainly consists of cellulose and sucrose, and its development results from sucrose decomposition and cellulose biosynthesis [6, 8]. Susy activity plays an important role in cellulose synthesis by providing the UDP-glucose substrate, which is essential for cell thickening and cotton fiber cell development [50, 51]. In developing cotton fibers (10, 15, 20 and 25 DPA), D13G2037 shows high and gradually increasing expression, but its expression in the long-fiber varieties was lower than that in the short-fiber varieties, and this difference approached statistical significance (Fig. 6d). This result might support the possibility that a certain range of D13G2037 expression promotes fiber elongation, whereas excessive expression might accelerate or alter the transition from the fast-elongation stage to the cell wall-thickening stage, resulting in a shorter fiber.

## Conclusions

This study provides the first developmental quantitative trait locus mapping of FL in upland cotton. A total of 15,369 SNP markers were genotyped in upland cotton using the Cotton 63 K Illumina SNP array, and these were employed in a GWAS of dynamic FL. In total, 21 QTLs related to dynamic FL were identified: seven of these have been verified in recent studies, and the other 14 QTLs were first identified in the present study. Two candidate genes, D10G1008 and D13G2037, were verified through qRT-PCR among four cultivars at the 10 to 25 DPA developmental stages of fiber, and these can be exploited to alter fiber development.

## Additional files

Additional file 1: **Table S1.** List of 83 upland accessions used for association mapping. P1 and P2 indicate the 83 upland cotton accessions grouped into two subpopulations by genotypes; the clustering indicates that the 83 upland cotton accessions were divided into two clusters by average growth rate trend of fiber length at the fast-elongation stage in the three years, and the letters A and B represent the cultivars in Fig. 1a and Fig. 1b, respectively. **Table S2.** List of 10 Sea Island cotton accessions. (XLSX 16 kb)

Additional file 2: **Figure S1.**. Frequency map of dynamic fiber length in upland cotton in different environments. (a) Fiber length at 10 DPA. (b) Fiber length at 15 DPA. (c) Fiber length at 20 DPA. (d) Fiber length at 25 DPA. (e) Fiber length at maturity. (TIF 1532 kb)

Additional file 3: **Figure S2.** Genome-wide association study (GWAS) of dynamic fiber length. The lowercase letters a through e represent Manhattan plots of the GLM at 10, 15, 20 and 25 DPA and maturity in 2014; f through l represent Manhattan plots of the GLM at 10, 15, 20 and 25 DPA and maturity in 2015; and m through q represent Manhattan plots of the GLM at 10, 15, 20 and 25 DPA and maturity in 2016, respectively. (PDF 893 kb)

Additional file 4: **Table S3.** Performance of backcross inbred lines (BILs) of Hai7124 × CRI36 hybrids and their parents. (XLSX 393 kb)

Additional file 5: **Figure S3.** Frequency map of fiber length at maturity in the BIL populations in three environments in 2016. (TIF 208 kb)

Additional file 6: **Table S4.** Genotype polymorphism of candidate SNPs between CRI36 and Hai 7124 and the primers used for HRM analysis. (XLSX 359 kb)

Additional file 7: **Table S5.** Correlation coefficients between SNP markers and FL at maturity in CRI36 × Hai 7124 BILs. (XLSX 9 kb)

Additional file 8: **Figure S4.** Gene Ontology (GO) analysis of 1221 candidate genes. (TIF 2974 kb)

Additional file 9: **Table S6.**. KEGG analysis of all candidate genes associated with dynamic fiber length. (XLSX 14 kb)

Additional file 10: **Figure S5.** Transcription profiles of differences in gene expression in fibers at 10 DPA between long- and short-fiber varieties. (TIF 2855 kb)

## Abbreviations

AGR: Average growth rate of fiber length
BILs: Backcross inbred lines
DPA: Days post anthesis
FL: Fiber length
GWAS: Genome-wide association study
HRM: High-resolution melting
LD: Linkage disequilibrium
MAF: Minor allele frequency
QTL: Quantitative trait loci; SNP: Single nucleotide polymorphism
