# Supplementary figures and images for: Genetic variation of dynamic fiber elongation and developmental quantitative trait locus mapping of fiber length in upland cotton (Gossypium hirsutum L.)
Source: BMC Genomics. 2018 Dec 6;19:882. doi: 10.1186/s12864-018-5309-2 (PMC6282333; doi:10.1186/s12864-018-5309-2)

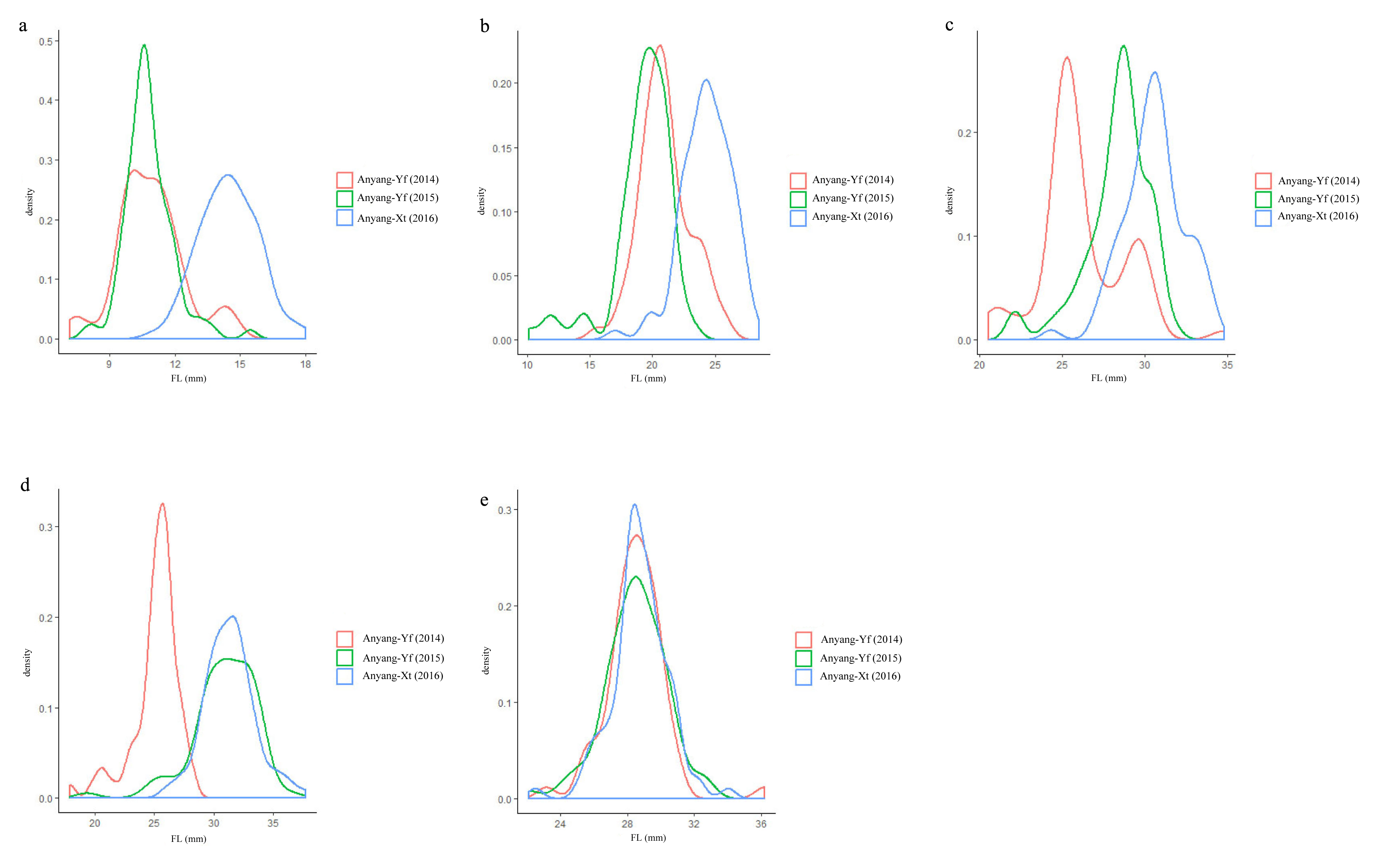

Supplement: Supplementary file 2 — Figure S1.. Frequency map of dynamic fiber length in upland cotton in different environments. (a) Fiber length at 10 DPA. (b) Fiber length at 15 DPA. (c) Fiber length at 20 DPA. (d) Fiber length at 25 DPA. (e) Fiber length at maturity. (TIF 1532 kb) [file 12864_2018_5309_MOESM2_ESM.tif]

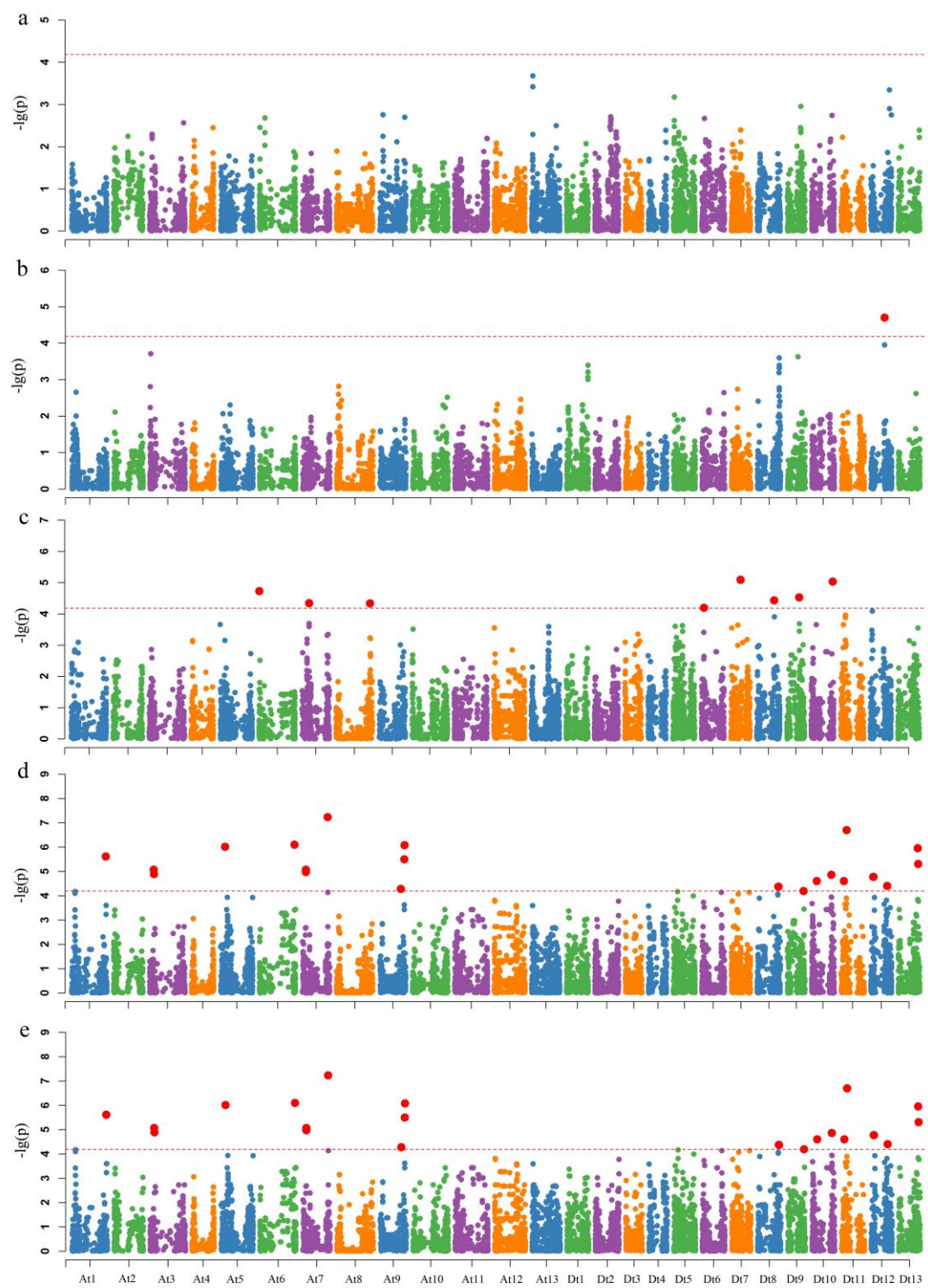

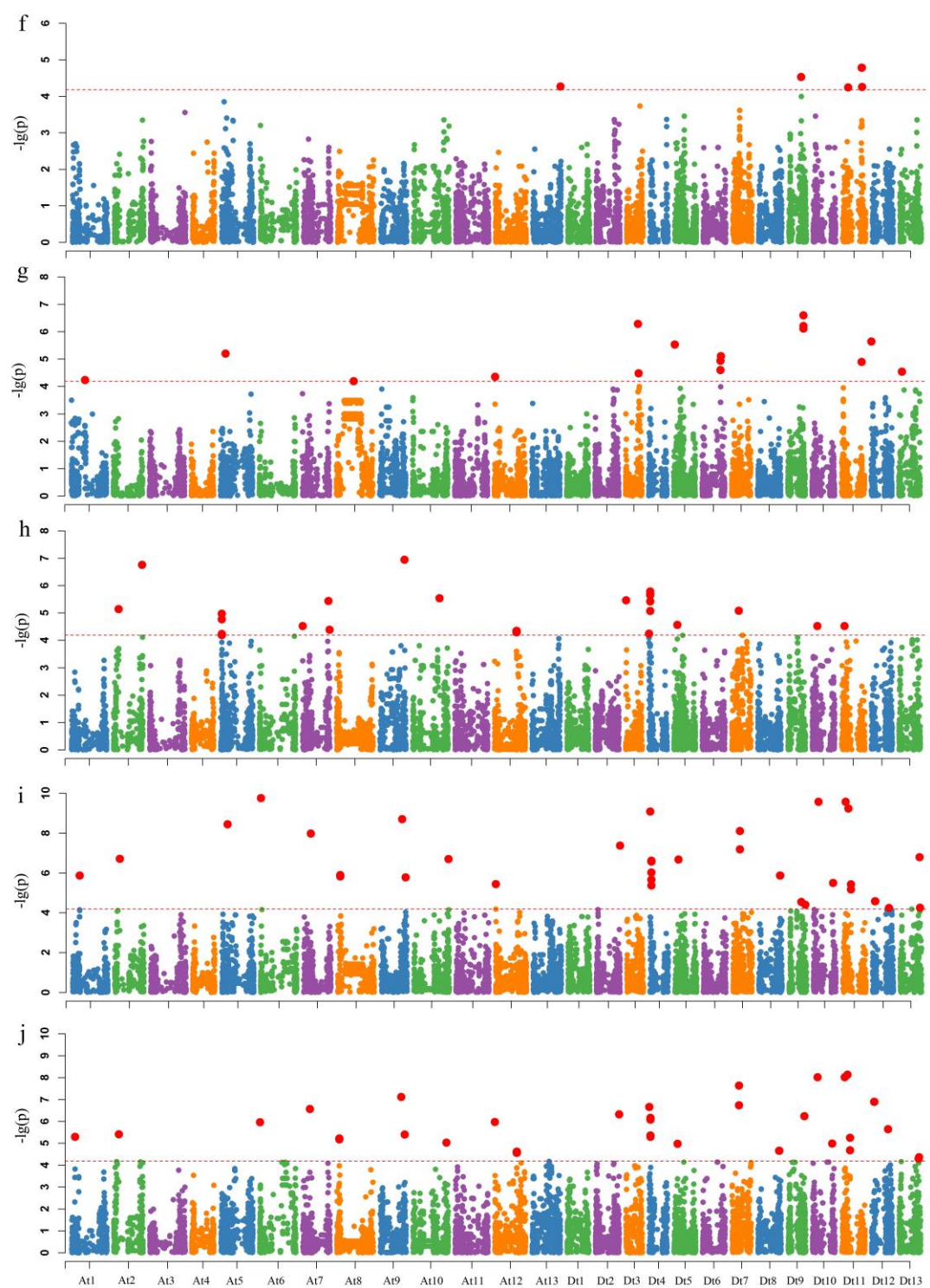

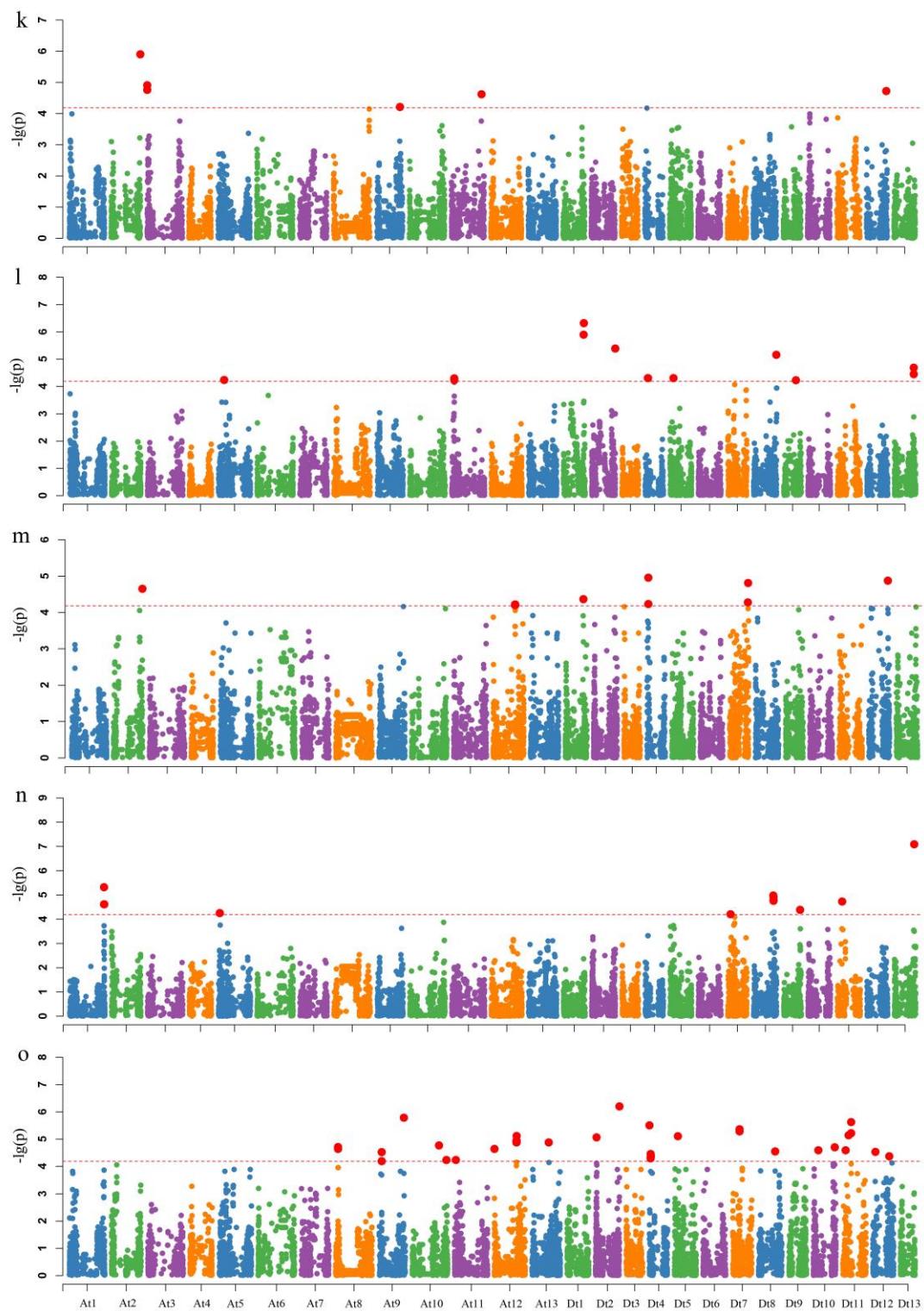

Supplement: Supplementary file 3 — Figure S2. Genome-wide association study (GWAS) of dynamic fiber length. The lowercase letters a through e represent Manhattan plots of the GLM at 10, 15, 20 and 25 DPA and maturity in 2014; f through l represent Manhattan plots of the GLM at 10, 15, 20 and 25 DPA and maturity in 2015; and m through q represent Manhattan plots of the GLM at 10, 15, 20 and 25 DPA and maturity in 2016, respectively. (PDF 893 kb) [file 12864_2018_5309_MOESM3_ESM.pdf]

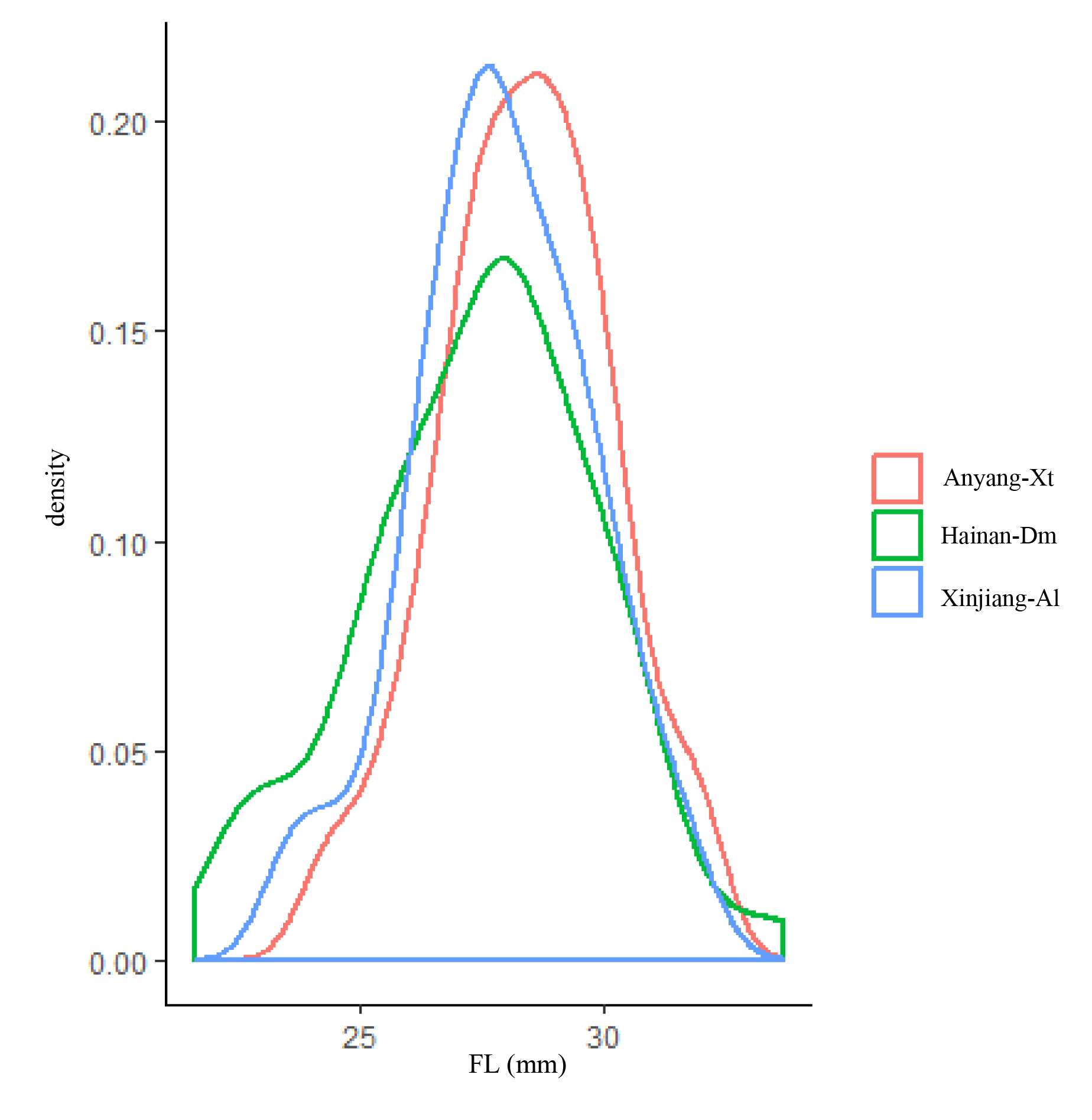

Supplement: Supplementary file 5 — Figure S3. Frequency map of fiber length at maturity in the BIL populations in three environments in 2016. (TIF 208 kb) [file 12864_2018_5309_MOESM5_ESM.tif]

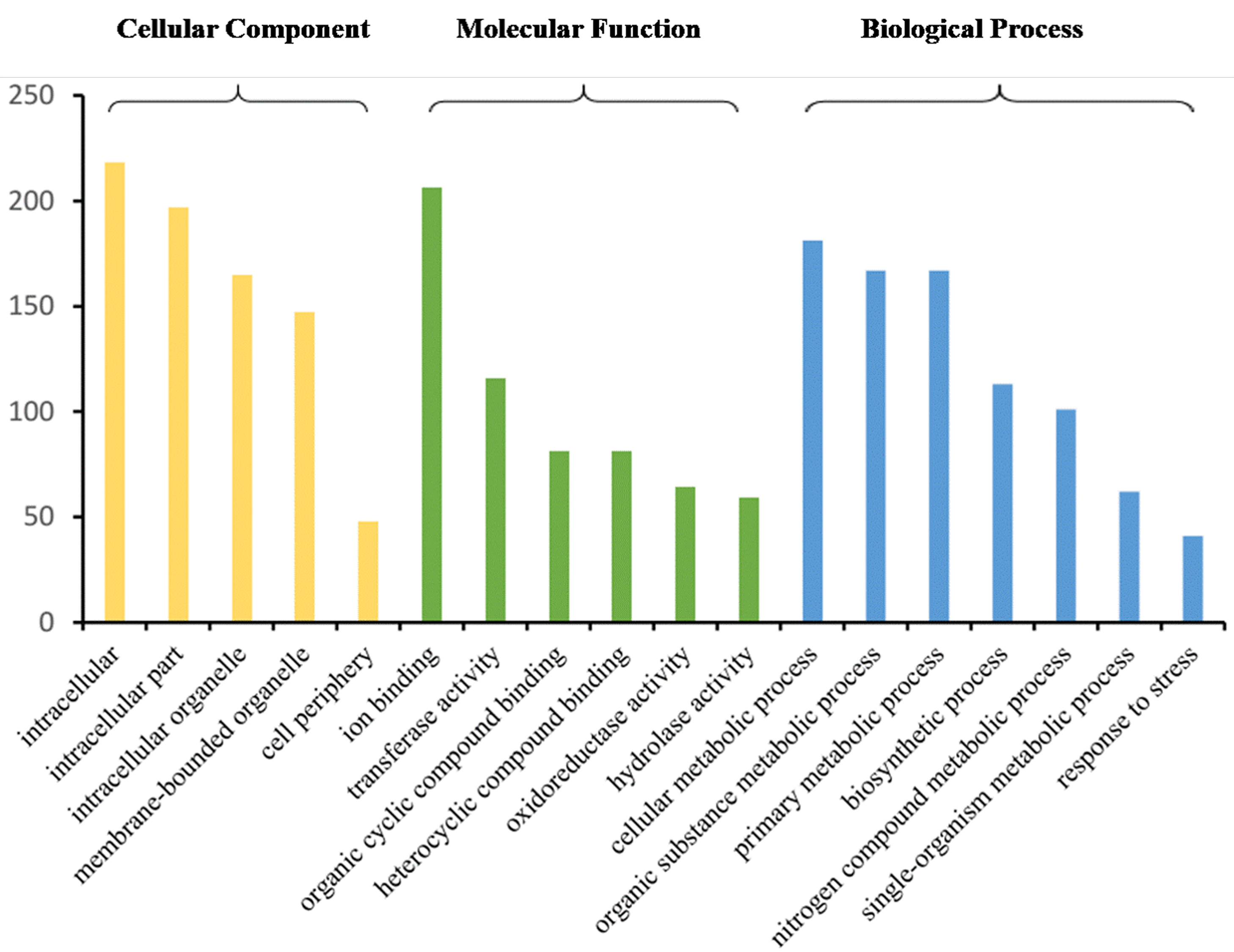

Supplement: Supplementary file 8 — Figure S4. Gene Ontology (GO) analysis of 1221 candidate genes. (TIF 2974 kb) [file 12864_2018_5309_MOESM8_ESM.tif]

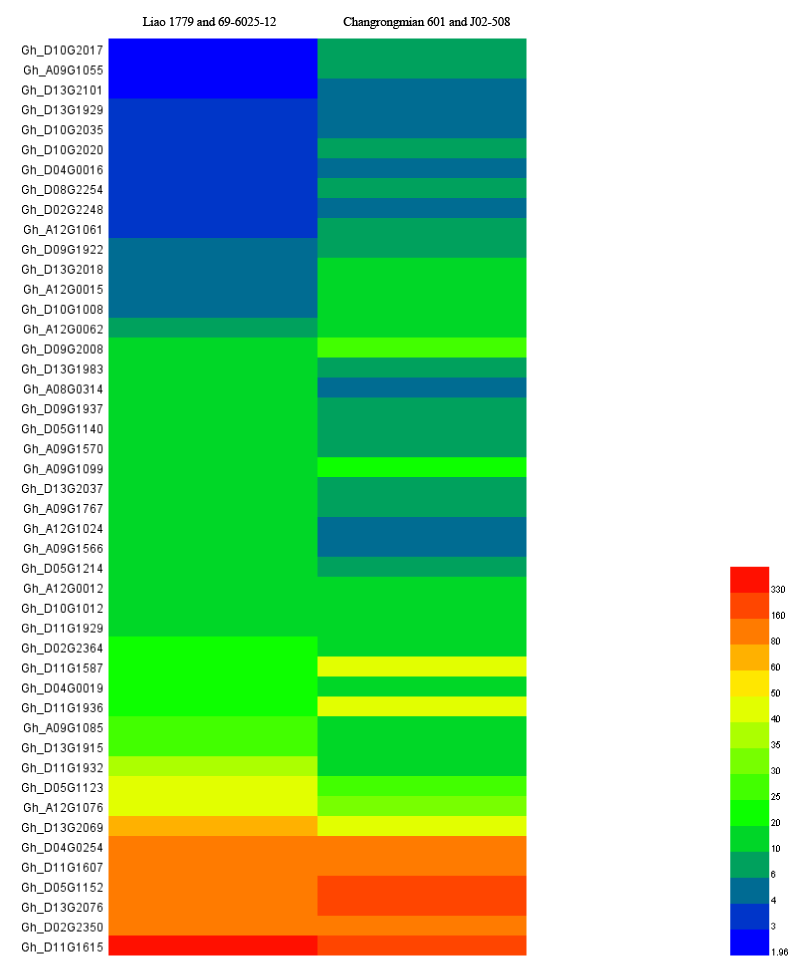

Supplement: Supplementary file 10 — Figure S5. Transcription profiles of differences in gene expression in fibers at 10 DPA between long- and short-fiber varieties. (TIF 2855 kb) [file 12864_2018_5309_MOESM10_ESM.tif]
